# Identification and inhibition of PIN1-NRF2 protein–protein interactions through computational and biophysical approaches

**DOI:** 10.1038/s41598-025-89342-0

**Published:** 2025-03-14

**Authors:** Adem Ozleyen, Gizem Nur Duran, Serhat Donmez, Mehmet Ozbil, Richard G. Doveston, Tugba Boyunegmez Tumer

**Affiliations:** 1 Leicester Institute for Structural and Chemical Biology, University of Leicester, Leicester, LE1 7RH UK; 2 School of Chemistry, University of Leicester, Leicester, LE1 7RH UK; 3 Health Institutes of Türkiye, Türkiye Biotechnology Institute, 06270 Ankara, Turkey; 4 Institute of Biotechnology, Gebze Technical University, 41400 Gebze, Kocaeli Turkey; 5 Graduate Program of Molecular Biology and Genetics, School of Graduate Studies, Canakkale Onsekiz Mart University, 17020 Canakkale, Turkey; 6 Institute of Science and Technology Austria (ISTA), 3400 Klosterneuburg, Austria; 7 Department of Molecular Biology and Genetics, Faculty of Arts and Science, Canakkale Onsekiz Mart University, 17020 Canakkale, Turkey; 8 Department of Medical Biotechnology, Faculty of Biochemistry, Biophysics and Biotechnology, Jagiellonian University, Kraków, Poland

## Abstract

NRF2 is a transcription factor responsible for coordinating the expression of over a thousand cytoprotective genes. Although NRF2 is constitutively expressed, its stability is modulated by the redox-sensitive protein KEAP1 and other conditional binding partner regulators. The new era of NRF2 research has highlighted the cooperation between NRF2 and PIN1 in modifying its cytoprotective effect. Despite numerous studies, the understanding of the PIN1-NRF2 interaction remains limited. Herein, we described the binding interaction of PIN1 and three different 14-mer long phospho-peptides mimicking NRF2 protein using computer-based, biophysical, and biochemical approaches. According to our computational analyses, the residues positioned in the WW domain of PIN1 (Ser16, Arg17, Ser18, Tyr23, Ser32, Gln33, and Trp34) were found to be crucial for PIN1-NRF2 interactions. Biophysical FP assays were used to verify the computational prediction. The data demonstrated that Pintide, a peptide predominantly interacting with the PIN1 WW-domain, led to a significant reduction in the binding affinity of the NRF2 mimicking peptides. Moreover, we evaluated the impact of known PIN1 inhibitors (juglone, KPT-6566, and EGCG) on the PIN1-NRF2 interaction. Among the inhibitors, KPT-6566 showed the most potent inhibitory effect on PIN1-NRF2 interaction within an IC_50_ range of 0.3–1.4 µM. Furthermore, our mass spectrometry analyses showed that KPT-6566 appeared to covalently modify PIN1 via conjugate addition, rather than disulfide exchange of the sulfonyl-acetate moiety. Altogether, such inhibitors would also be highly valuable molecular probes for further investigation of PIN1 regulation of NRF2 in the cellular context and potentially pave the way for drug molecules that specifically inhibit the cytoprotective effects of NRF2 in cancer.

**Supplementary Information:**

The online version contains supplementary material available at 10.1038/s41598-025-89342-0.

## Introduction

In 2024, we celebrated the 30th anniversary of the discovery of nuclear factor erythroid 2-related factor 2 (NRF2)^1^ Over the years, investigations in this field have underscored the crucial roles of NRF2, a transcription factor that regulates cytoprotective genes to mitigate various pathologies relating to inflammatory, neurodegenerative, respiratory, and cardiovascular diseases. When cells are exposed to oxidative stress, NRF2 is activated and translocates to the nucleus where it binds to antioxidant response elements (AREs) in the DNA, triggering the expression of various cytoprotective genes encoding antioxidant and phase II detoxification enzymes and other cytoprotective proteins. In 1997, NRF2 was known to orchestrate the expression of more than 1000 cytoprotective genes^2^. By 2023, however, there was evidence that about 2000 genes could be directly or indirectly regulated by NRF2^3^. Overall, such a large number of genes, representing more than 1% of the human genome, interacting between redox homeostasis and NRF2 activation is essential for cellular health and resilience to oxidative stressors^4^. NRF2 is constitutively expressed but dynamically regulated at the protein level by the redox-sensitive protein KEAP1 (Kelch-like ECH-associated protein 1)^5^. A large body of research has detailed the repressive function of KEAP1 in intricately fine-tuning the activity of NRF2 through a direct binding partnership^6,7^.

NRF2 is also regulated by other conditional binding partner proteins that function as either corepressors (SMRT, GR, RXRα, and β-TrCP), or coactivators (CBP, MED16, BRG1, and RAC3)^8^. The new era of NRF2 research has also highlighted the role of peptidyl-prolyl cis–trans isomerase PIN1 in stabilising NRF2 and modulating its cytoprotective capacity. PIN1 is a peptidyl-prolyl isomerase (PPIase) that catalyzes the cis–trans isomerization of substrate proteins via specific recognition of phosphorylated serine-proline and/or threonine-proline (pS/pT-P) motifs^9^. It is a relatively small protein consisting of an N-terminal WW domain, a phosphoprotein binding module and a C-terminal catalytic domain with isomerase activity (Supplementary Fig. 1b). PIN1 activity plays an important role in regulating the function of over 200 substrate proteins involved in regulating the cell cycle, cell motility, apoptosis, and neuronal differentiation^10^. Dysregulation of PIN1 is associated with the development of various pathologies including cancer, obesity, diabetes, and inflammation. For example, PIN1 overexpression was found to increase NRF2 ubiquitination, resulting in ROS-mediated vascular smooth muscle cell proliferation through the down-regulation of NRF2/ARE-dependent heme oxygenase-1 (HO-1) protein expression^11^. Additionally, PIN1- deficient mouse embryonic fibroblast cells were more resistant to oxidative stress due to upregulation of NRF2 downstream antioxidant genes involving NAD(P)H Quinone Dehydrogenase 1(NQO1) and Glutathione S-transferase A1 (GSTA1)^12^.

In contrast, recent studies have highlighted the importance of NRF2 stabilization/activation by PIN1 in cancer progression, providing new insights into the underlying mechanisms. It has been proposed that NRF2 and PIN1 cooperate to modify the cytoprotective potential of NRF2. Through this mechanism, cancer cells enhance their antioxidant capacity and evade apoptotic and ferroptotic cell death. In the study of Liang et al.^13^ it was suggested that PIN1 stabilizes NRF2 and enhances its activity in pancreatic ductal carcinoma with high K-Ras activity. This led to increased expression of antioxidant genes, maintenance of redox balance and counteracting mitochondrial respiratory injury induced by K-Ras. Therefore, the study proposes that targeting the PIN1/c-Myc/NRF2 axis may be a potential therapeutic strategy for the treatment of pancreatic cancer. Additionally, the study of Zhang et al.^14^ demonstrated that PIN1 silencing in cervical cancer cells decreases the stability and activity of NRF2, thereby downregulation of Glutathione Peroxidase 4 (GPX4), resulting in increased cellular susceptibility to cisplatin-induced oxidative stress, ferroptosis, and apoptosis. Moreover, the studies on breast cancer cells highlighted that H-Ras induces an interaction between NRF2 and PIN1 leading to increased expression of genes involved in antioxidant defence and cancer progression. Furthermore, PIN1 knockdown in breast cancer cells resulted in a decrease in NRF2 protein levels and activity, indicating that PIN1 is required for the stability and activation of NRF2^15^. The same research group assessed the potential binding sites within NRF2 that interact with PIN1^16^. Based on co-immunoprecipitation assays, NRF2 phosphorylation at S215, S408, and S577 by MAPKs was essential for the PIN1-NRF2 protein–protein interactions (PPIs) in a triple-negative breast cancer cell line where K-Ras is naturally overexpressed. These residues fall within the NRF2 Neh7 domain, Neh6-Neh1 linker region, and Neh 3 domain respectively, all of which are important for different elements of NRF2 function.

**Fig. 1:**
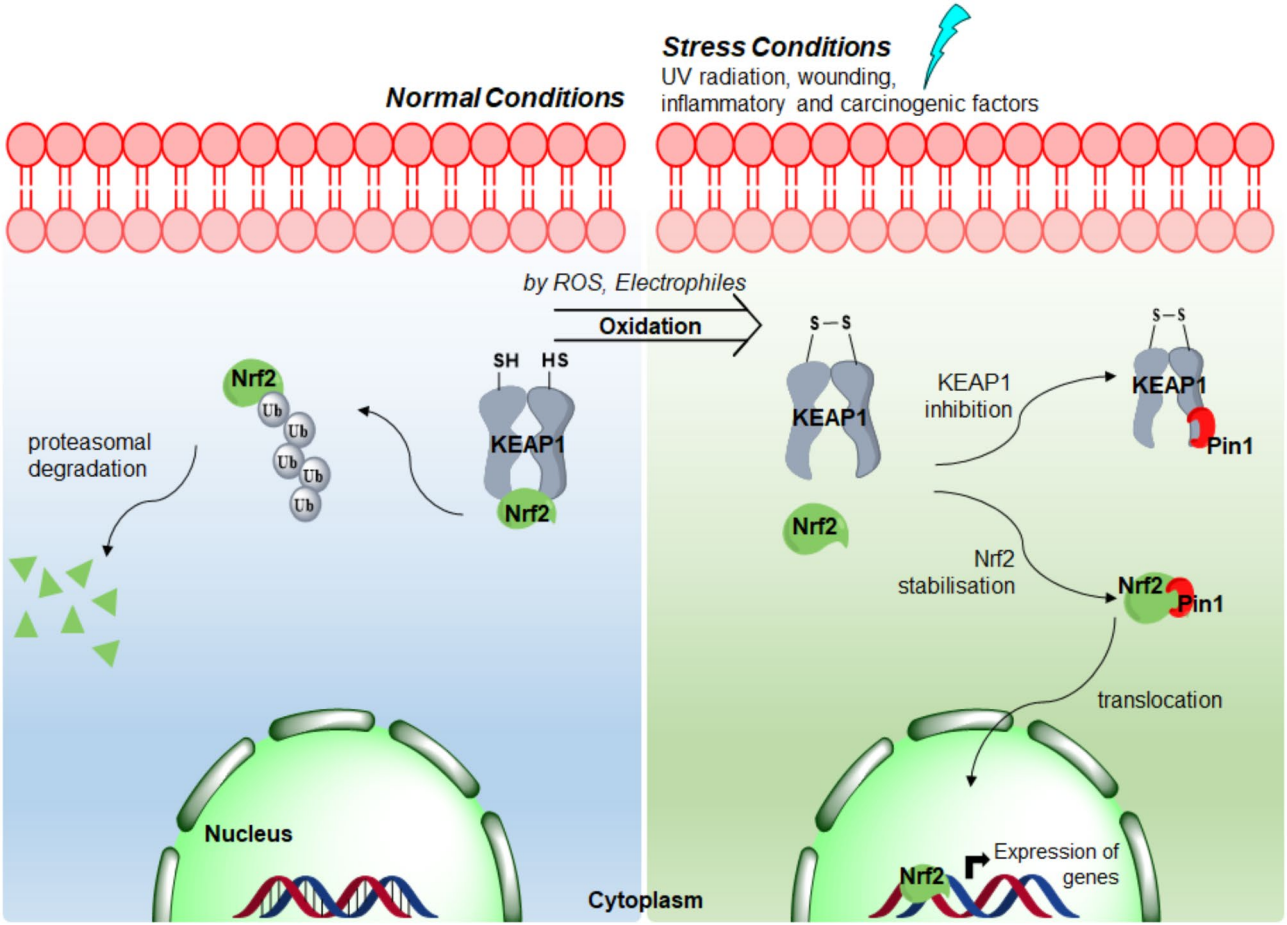
The proposed mechanism of intracellular NRF2 modulation by KEAP1 and PIN1 proteins.

Saeidi et al.^16^ also reported an alternative mechanism of NRF2 stabilization involving PIN1-mediated sequestration of NRF2. This is an indirect effect resulting from PIN1 binding to the BTB and IVR domains of KEAP1 which impedes KEAP1-mediated NRF2 degradation. This indirect mechanism increases NRF2 protein activity and provides a novel means of NRF2 regulation^16^(Fig. 1).

Based on these findings, we aimed to investigate the PIN1-NRF2 interaction using an integrated computational and biophysical approach. Specifically, we aimed to: (i) confirm the phosphorylated NRF2 peptide motifs that interact with PIN1, and determine their binding energies using molecular simulation analyses; (ii) provide qualitative data on the affinity of the interactions and; (iii) assess the effects of PIN1 inhibitors on these specific interactions. Additionally, we sought to identify the residues and domains of PIN1 that were essential for the PIN1-NRF2 interaction with a view to identifying druggable target sites for specific NRF2-PIN1 interactions.

## Results

### Biophysical and computational approach

Phosphorylation of NRF2 at S215, S408, and S577 is known to be essential for the interaction with PIN1. Interpretations of experimental data and computational analyses have shown that NRF2 exhibits the characteristic features of intrinsically disordered proteins (IDPs)^17^ (Supplementary Fig. 1a). Therefore, despite the crucial role of NRF2 in the maintenance of redox homeostasis, its 3D structure has not yet been fully determined. Of the seven domains, the structures of the Neh2 from residues M1 to G98 and part of the Neh1 DNA-binding domain from residues K445 to V523 were identified^18^. The Neh 4 and Neh 5 domains also have structural elements. However, while the Neh 6 and Neh 3 domains are classified as predominantly unstructured, the Neh1, Neh2 and Neh 7 domains are partially unstructured^19^. Therefore, elucidating the physical interactions of NRF2 with different binding partners requires a multi-faceted approach. In the current work, we have combined bioinformatics tools with biophysical and biochemical approaches.

Three 14 amino acid synthetic phospho-peptides, each mimicking a different phosphorylated NRF2 motif, were used to study the putative PIN1-NRF2 PPIs: NRF2_209-222_^pSer215^, NRF2_402-415_^pSer408^, and _NRF2571-584_^pSer577^ (Supplementary Fig. 2). All peptides contained a carboxylate C-terminus, and either acetylation or fluorescein labelling via an aminohexanoic acid linker (Ahx) at the N-terminus.

**Fig. 2:**
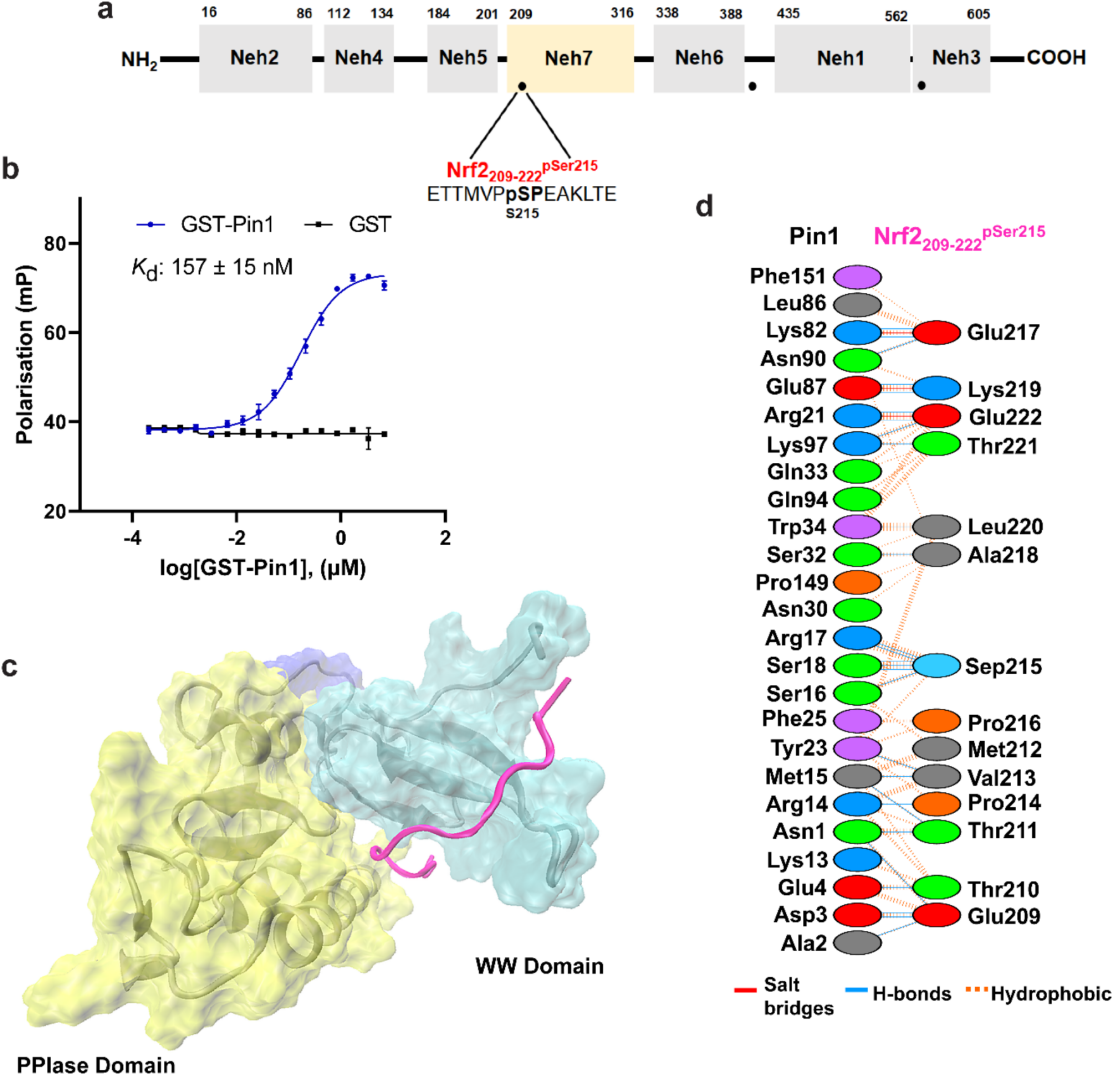
NRF2_209-222_^pSer215^ phospho-peptide interaction with PIN1. (**a**) The position of NRF2_209-222_^pSer215^ within NRF2. (**b**) FP data to assess the binding affinity of NRF2_209-222_^pSer215^ with GST-PIN1 (blue curve). GST-PIN1 was titrated to fluorescently labelled NRF2_209-222_^pSer215^ peptide (10 nM). GST protein was used as a control (black line). Error bars represent standard deviation for n = 3 replicates. (**c**) The binding position of NRF2_209-222_^pSer215^ observed in molecular simulation analyses. (**d**) Diagram showing the interactions between PIN1 and NRF2_209-222_^pSer215^ at the amino acid residue level.

**Fig. 3:**
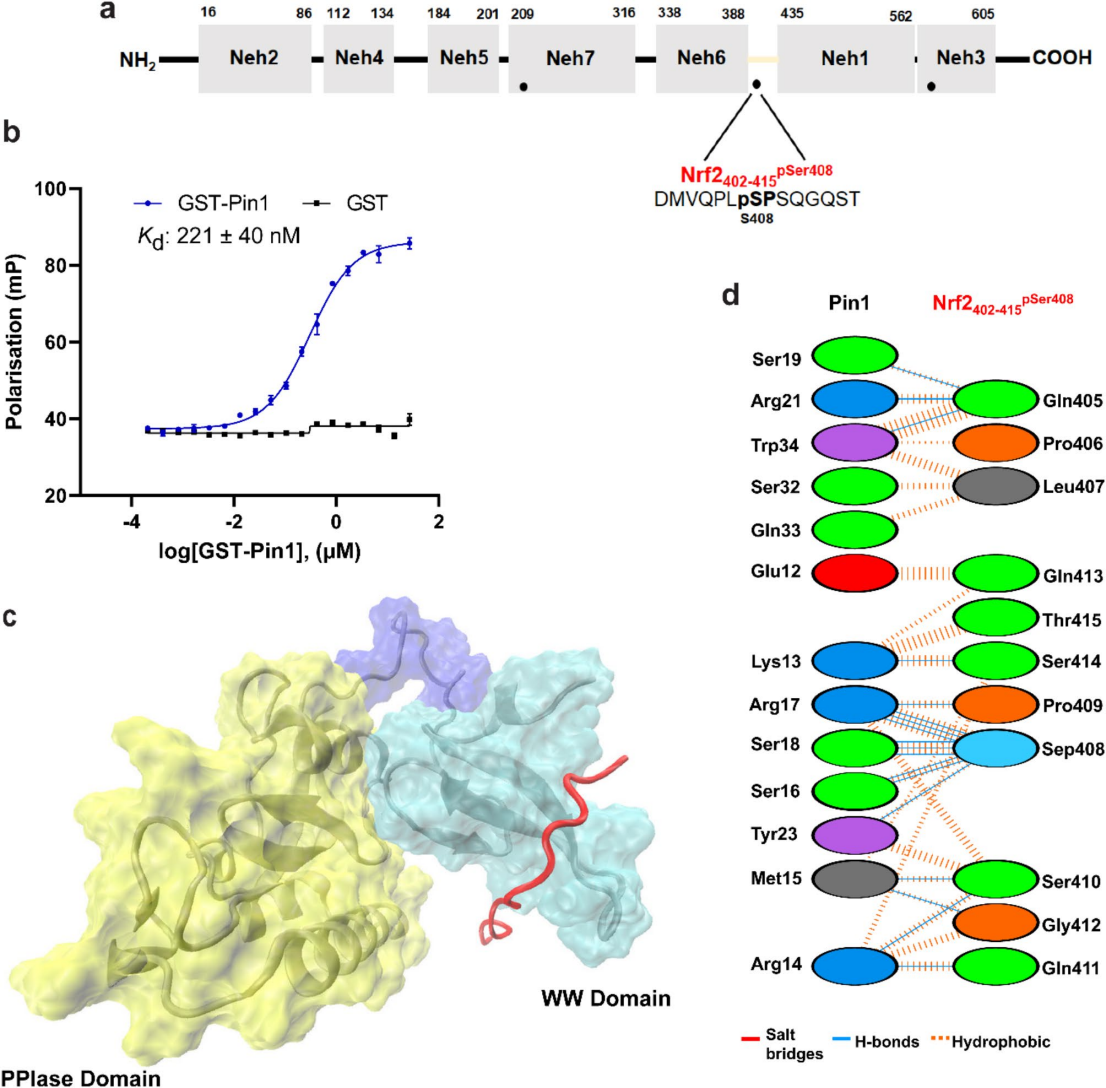
NRF2_402-415_^pSer408^ phospho-peptide interaction with PIN1. (**a**) The position of NRF2_402-415_^pSer408^ within NRF2. (**b**) FP data to assess the binding affinity of NRF2_402-415_^pSer408^ with GST-PIN1 (blue curve). GST-PIN1 was titrated to fluorescently labelled NRF2_402-415_^pSer408^ peptide (10 nM). GST protein was used as a control (black line). Error bars represent standard deviation for n = 3 replicates. (**c**) The binding position of NRF2_402-415_^pSer408^ observed in molecular simulation analyses. (**d**) Diagram showing the interactions between PIN1 and NRF2_402-415_^pSer408^ at the amino acid residue level.

**Fig. 4:**
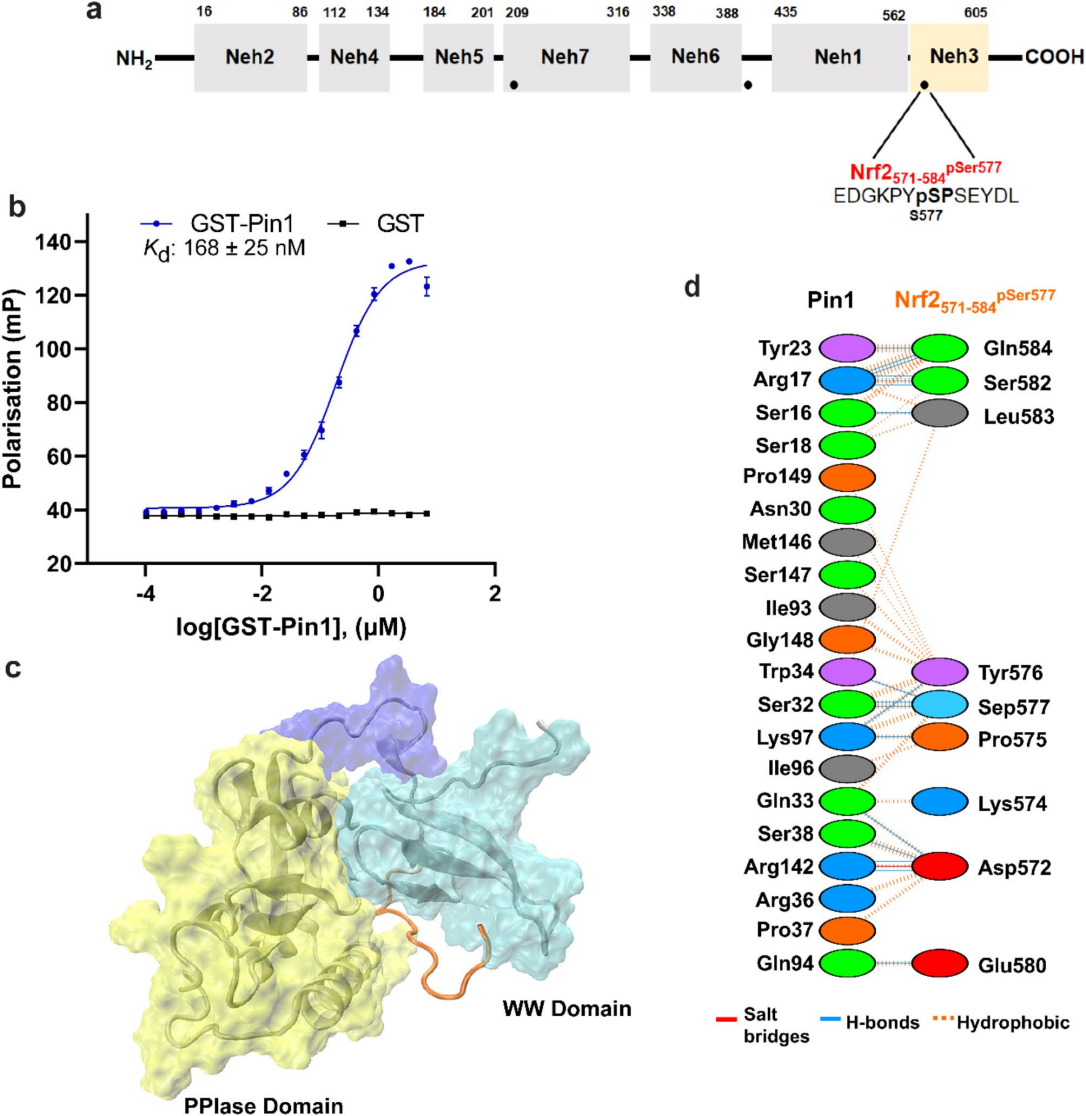
NRF2_571-584_^pSer577^ phospho-peptide interaction with PIN1. (**a**) The position of NRF2_571-584_^pSer577^ within NRF2. (**b**) FP data to assess the binding affinity of NRF2_571-584_^pSer577^ with GST-PIN1 (blue curve). GST-PIN1 was titrated to fluorescently labelled NRF2_571-584_^pSer577^peptide (10 nM). GST protein was used as a control (black line). Error bars represent standard deviation for n = 3 replicates. (**c**) The binding position of NRF2_571-584_^pSer577^observed in molecular simulation analyses. (**d**) Diagram showing the interactions between PIN1 and NRF2_571-584_^pSer577^ at the amino acid residue level.

The relative binding affinities of the phospho-peptides for PIN1 were determined using fluorescence polarisation (FP) assays. The three PIN1 constructs (PIN1, HIS_6_-PIN1, and GST-PIN1) were titrated to a fixed concentration of the fluorescently labelled NRF2-mimicking phospho-peptides, and a previously reported non-native PIN1 peptidic ligand, ‘Pintide’ (10 nM). Pintide is a synthetic phosho-peptide that was optimised for high affinity recognition by the PIN1 WW-domain^20,21^. A variable slope dose–response model was used to fit the data and obtain apparent dissociation constants (*K*_d_) for the interactions. Titration of PIN1 and HIS_6_-PIN1 did not yield suitable binding curves, likely due to a relatively small size difference between the protein construct and the phospho-peptide fluorescent tracers (Supplementary Fig. 3). Titration of the larger GST-tagged PIN1 construct did result in sigmoidal binding curves, and the apparent *K*_d_ of the reference Pintide was found to be 17 ± 3 nM, which was consistent with literature values (Supplementary Fig. 4)^20,21^. GST protein alone did not result in any change in polarisation indicating that the interactions were specific to PIN1 (Figs. 2b, 3b, 4b). GST-PIN1 was therefore used for the FP binding experiments because its larger size facilitated a more favourable assay window. However, the exact influence of the GST tag on PIN1 structure and the apparent binding affinity requires further investigation. The sequences of unlabelled NRF2 mimicking phospho-peptides were employed for molecular docking and molecular dynamics simulations to reveal their binding sites and highlight intermolecular non-covalent interactions between NRF2_209-222_^pSer215^-PIN1, NRF2_402-415_^pSer408^-PIN1, and NRF2_571-584_^pSer577^-PIN1, respectively.

### Interaction of NRF2_209-222_^pSer215^ peptide with PIN1

Titration of GST-PIN1 to the fluorescently labelled NRF2_209-222_^pSer215^ peptide (10 nM, Fig. 2a) led to a sigmoidal binding curve from which the apparent dissociation constant for the interaction was determined as 157 ± 15 nM (Fig. 2b). This interaction was almost tenfold weaker than the optimised Pintide ligand, but nevertheless had significant and physiologically relevant affinity for PIN1. According to the computational calculations, the NRF2_209-222_^pSer215^ peptide was located at the WW domain of PIN1 and the last four residues interacted with the PPIase domain. The interactions were a mixture of salt bridges, H-bonds, and hydrophobic interactions (Fig. 2c,d) (Supplementary Table 1), and all 14 residues of the peptide were involved in interactions. The N-terminal of the peptide interacted with PIN1 mainly through hydrophobic interactions, whereas the C-terminal interacted mainly through hydrophilic interactions.

Ser215 is located within the Neh7 domain of NRF2, also known as the RXRα/RARα binding domain. The Neh7 domain directly interacts with retinoic X receptor alpha (RXRα) resulting in suppression of NRF2 activity and a decrease in its cytoprotective effects^22^. Additionally, the heterodimer of RXRα and the retinoic acid receptor alpha (RARα) antagonises NRF2 activity^23^. Collectively, RXRα/RARα interactions with the NRF2 Neh7 domain prevent the binding of coactivators to the neighbouring Neh4 and Neh5 transactivation domains leading to loss of NRF2 activity. PIN1 could therefore protect or enhance NRF2 activity in response to phosphorylation by competing for binding to the Neh7 domain with the RXRα/RARα antagonists. This effect might be more pronounced in cancer cells where PIN1 expression levels and MAPK activity are high.

### Interaction of NRF2_402-415_^pSer408^ peptide with PIN1

Titration of GST-PIN1 to the fluorescently labelled NRF2_402-415_^pSer408^ peptide (10 nM, Fig. 3a) led to a sigmoidal binding curve from which the apparent dissociation constant for the interaction was determined as 221 ± 40 nM (Fig. 3b). This interaction was of comparable affinity to the NRF2_209-222_^pSer215^ peptide (157 nM), and again ~ 13-fold weaker than the optimised Pintide ligand. Based on the molecular simulation analyses, the NRF2_402-415_^pSer408^ peptide was located solely on the WW domain, and the interactions consisted of hydrophobic and hydrogen bonds (Fig. 3c,d) (Supplementary Table 2), which were evenly distributed along the peptide. According to the obtained pose, 11 residues of this peptide were found to be involved in these PPIs, in comparison to all 14 residues of the NRF2_209-222_^pSer215^ peptide.

Ser408 is located in a linker region between the NRF2 Neh6 and Neh1 domains, a region not previously associated with any PPI (Fig. 3a). It is most proximal (20 amino acids) to the Neh6 domain which consists of two separate binding sites for β-transducin repeat-containing protein (β-TrCP, ^343^DSGIS^347^ and ^382^DSAPGS^387^). Binding of β-TrCP leads to KEAP1-independent NRF2 degradation^24^. Therefore, PIN1 binding could protect or enhance NRF2 activity by disrupting β-TrCP binding. However, the potential competition between PIN1 and β-TrCP for NRF2 binding has not been investigated.

### Interaction of NRF2_571-584_^pSer577^peptide with PIN1

Titration of GST-PIN1 to the fluorescently labelled NRF2_571-584_^pSer577^ peptide (10 nM, Fig. 4a) led to a sigmoidal binding curve from which the apparent dissociation constant for the interaction was determined as 168 ± 25 nM (Fig. 4b). This interaction was slightly higher affinity compared to the other NRF2 phospho-peptide motifs, and again ~ tenfold weaker than the optimised Pintide ligand. The simulations showed that NRF2_571-584_^pSer577^ was buried in the cleft between PPIase and WW domains of PIN1 protein. Similar to PIN1- NRF2_209-222_^pSer215^ interactions, the interactions were a mixture of H-bonds, and hydrophobic interactions (Fig. 4c,d) (Supplementary Table 3). However, only 9 of 14 residues of the peptide were involved in interactions with PIN1.

Ser 577 is located in the Neh3 domain within the C-terminus of NRF2 (Fig. 4a). A motif within the Neh3 domain (_591_VFLVPK_596_) has been identified as a crucial binding site for recruiting chromodomain helicase DNA-binding domain protein 6 (CHD6) to facilitate its transcriptional activity^25^.

### Comparison of PIN1 binding sites on NRF2

The average calculated relative binding free energies for the three peptides were -12.49 kcal/mol, -7.05 kcal/mol, and -6.28 kcal/mol for NRF2_209-222_^pSer215^, NRF2_402-415_^pSer408^, and NRF2_571-584_^pSer577^, respectively (Supplementary Fig. 5). The error margin of the method was around 0.5 kcal/mol and the margin between NRF2_402-415_^pSer408^ and NRF2_571-584_^pSer577^ was just above this margin. Thus, NRF2_209-222_^pSer215^ was bound to PIN1 with significantly higher relative binding free energy than NRF2_402-415_^pSer408^ and NRF2_571-584_^pSer577^, which were bound to PIN1 with similar relative binding free energies. The significant difference between NRF2_209-222_^pSer215^ and other peptides were due to binding location of the peptide, in the cleft between the WW and PPIase domains, mainly interacting with the WW domain, and numerous intermolecular interactions of various kinds. These subtleties were not resolved by the FP data where the apparent *K*_d_ values for all three peptides were comparable, considering the calculated error. The FP data provides confirmation that all three peptides bind to PIN1, but because the technique reports on a global average of all binding events, it cannot differentiate subtle differences in the thermodynamic landscape.

### The PIN1 WW domain is crucial for interactions with NRF2

**Fig. 5:**
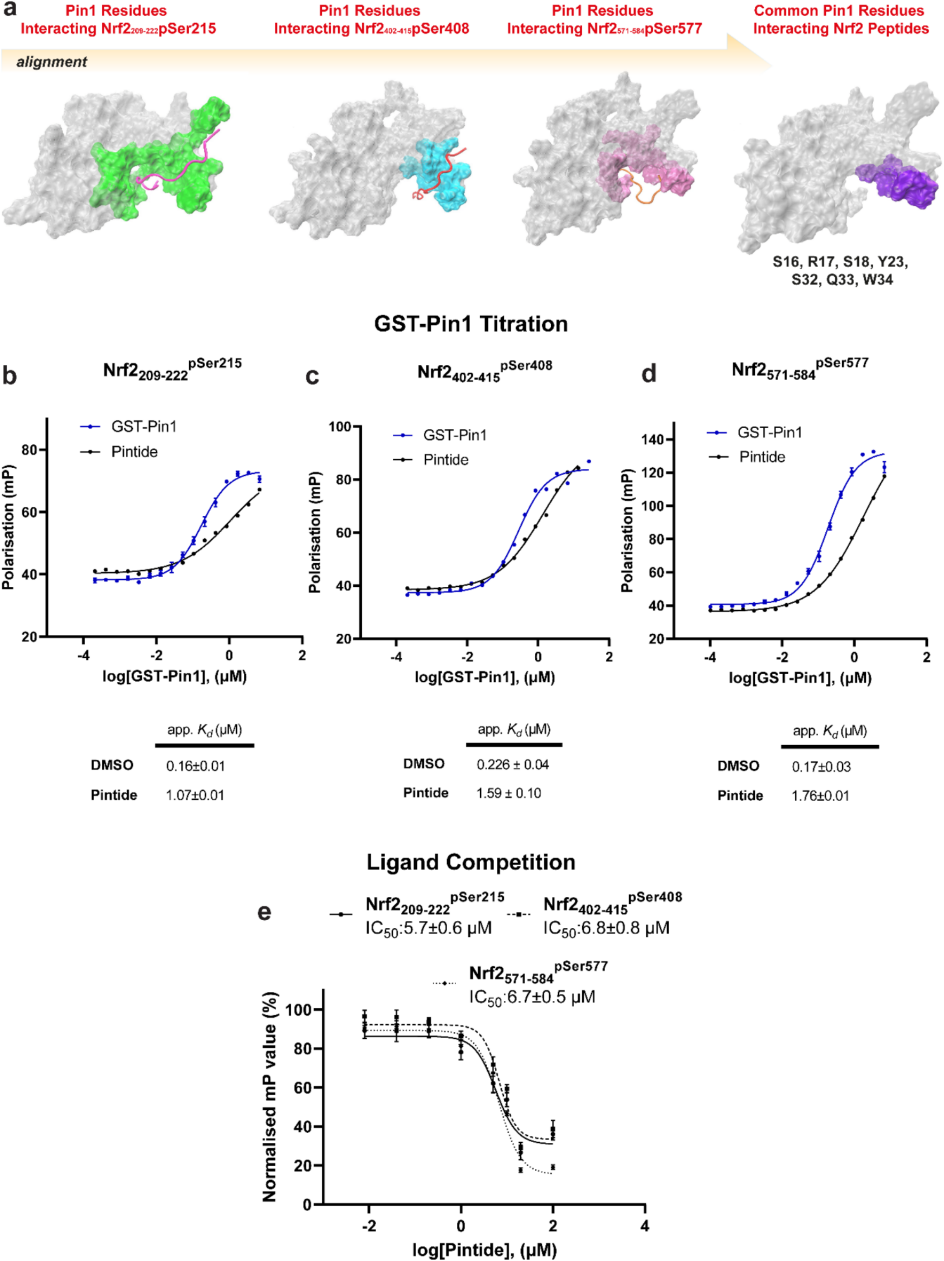
The role of WW domain for PIN1-NRF2 interactions. (**a**) Alignment of PIN1 proteins highlighting common interaction residues essential for NRF2 mimicking peptides. (**b**–**d**) FP experiments depict the titration of GST-PIN1 to NRF2_209-222_^pSer215^, NRF2_402-415_^pSer408^, and NRF2_571-584_^pSer577^ (10 nM) in the presence of DMSO and WW domain-selective Pintide (10 µM), respectively. (**e**) graphs illustrate the IC_50_ values of pintide, indicating the inhibition of NRF2 mimicking peptides, respectively. Data, collected from three independent experiments, are presented as mean ± standard error of the mean.

According to the commonly accepted mode of interaction of the PIN1 protein with its substrates, the WW domain is responsible for substrate recognition and specificity, while the PPIase domain is responsible for the catalytic activity^26^. In vitro binding assays have suggested that the WW domain has a ten times higher affinity for its pSP binding motif compared to the PPIase domain^20,27^. The results of our study align with this dogma: molecular simulations predict that all NRF2 mimicking phospho-peptides interact with the WW domain (Fig. 5a). The NRF2_209-222_^pSer215^ and NRF2_571-584_^pSer577^ peptides also interacted with the interface between the WW- and PPIase-domains, whereas the NRF2_402-415_^pSer408^ peptide was solely bound to the WW domain. The PIN1 linker region, and domain-domain interface contacts are important features of PIN1 interdomain allostery, structure and function, which are known to be heavily influenced by substrate binding^28^. Thus, our computational results comply with the hypothetical mode of interactions.

To determine the common interacting site at the PIN1 WW-domain, the simulated PIN1-NRF2 peptide complexes were aligned. The PIN1 residues that interacted with all NRF2 phospho-peptides were Ser 16, Arg 17, Ser 18, Tyr 23, Ser 32, Gln 33, and Trp 34 (Fig. 5a). This was consistent with other studies on the PIN1-Cdc25C^14^ and RNA polymerase C-terminal domain (CTD)^29,30^.

FP assays were used to verify the computational prediction that the NRF2 phospho-peptides predominantly interacted with the PIN1 WW-domain. We hypothesised that because Pintide has selectivity for the PIN1 WW-domain it should impede NRF2 phospho-peptide binding. First, GST-PIN1 was titrated to the fluorescently labelled Nrf 2 phospho-peptides (10 nM) in the presence of a fixed concentration of Pintide (10 µM). The presence of Pintide led to a 7-, 7-, and tenfold reduction in binding affinity of the fluorescently labelled NRF2_209-222_^pSer215^, NRF2_402-415_^pSer408^, and NRF2_571-584_^pSer577^ NRF2-phospho peptides respectively (Fig. 5b–d). However, none of the curves reached a saturation point meaning the apparent *K*_d_ values obtained are estimated values.

Next, a competition binding assay was used to determine if Pintide competed for PIN1 binding with the NRF2 peptides in a concentration dependent manner. Thus, Pintide was titrated to a fixed concentration of GST-PIN1 (1 µM) and fluorescently labelled NRF2 peptide (10 nM). In each case this led to Pintide concentration dependent decrease in polarisation, indicating that Pintide did indeed compete for binding with IC_50_ values of 5.7, 6.8, and 6.7 µM for the NRF2_209-222_^pSer215^, NRF2_402-415_^pSer408^, and NRF2_571-584_^pSer577^ phospho-peptides respectively (Fig. 5e). Together, the computational analysis and FP assays provide a strong indication that the phosphorylated NRF2 peptide mimics interact with PIN1 via its WW-domain.

### Small molecule inhibition of PIN1-NRF2 interactions

**Fig. 6:**
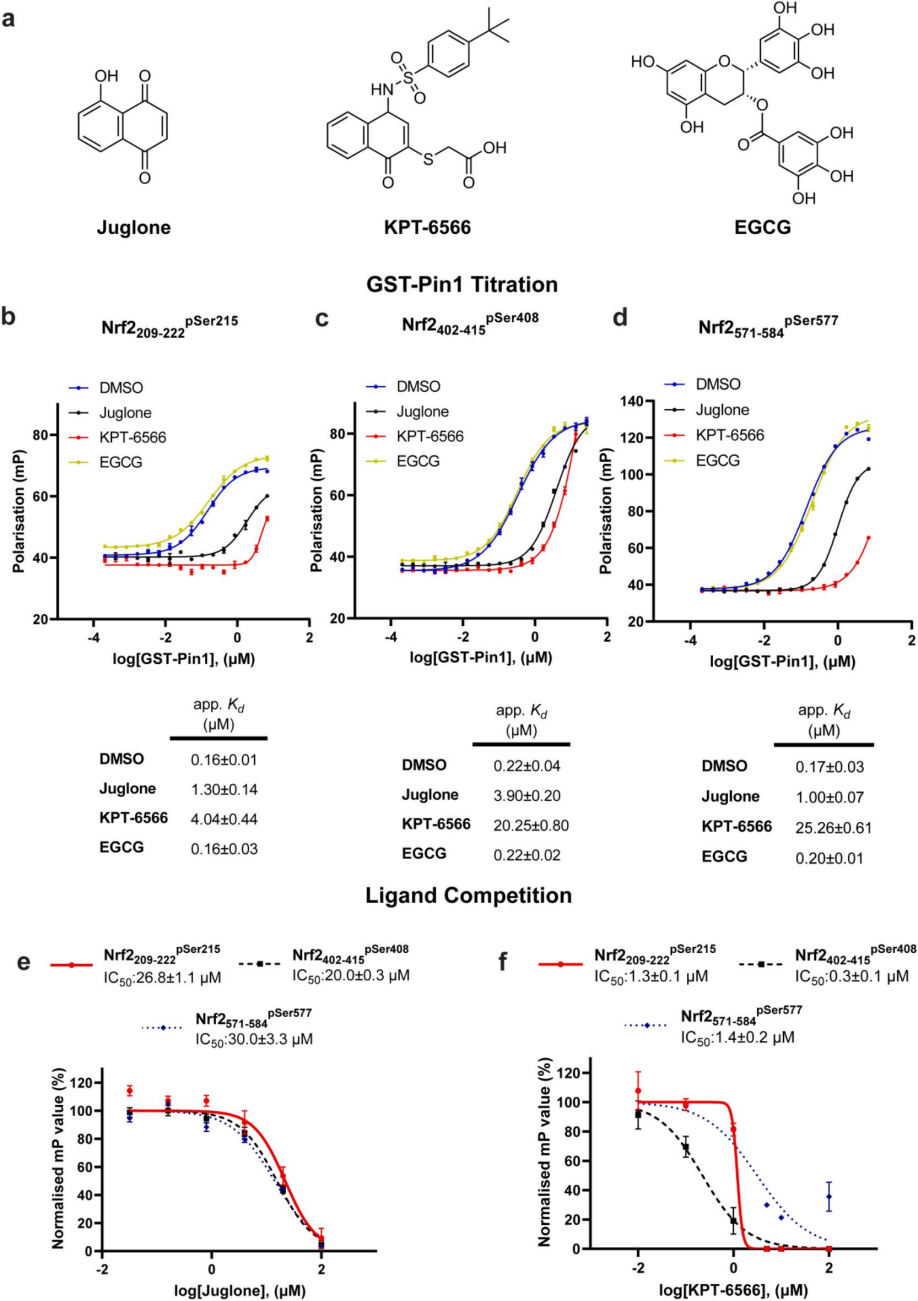
Inhibitory properties of PIN1 inhibitors on binding of PIN1-NRF2 PPI. (**a**) The structures of PIN1 inhibitors; juglone, KPT-6566, and EGCG. (**b**–**d**) FP experiments depict the titration of GST-PIN1 to NRF2_209-222_^pSer215^, NRF2_402-415_^pSer408^, and NRF2_571-584_^pSer577^ (10 nM) in the presence of DMSO and inhibitors (100 µM), respectively. (**e**, **f**) The IC_50_ values of juglone and KPT-6566 were determined for the inhibition of NRF2 mimicking NRF2_209-222_^pSer215^, NRF2_402-415_^pSer408^, and NRF2_571-584_^pSer577^ peptides, respectively. Data, obtained from three independent experiments, are presented as mean ± standard error of the mean.

Although there are currently not clinically approved PIN1 selective inhibitors, there are a variety of small compounds under in vitro-based investigation for their PIN1 inhibitory properties. In this study, we integrated three different PIN1 inhibitors, including juglone, epigallocatechin gallate (EGCG), and KPT-6566 (Fig. 6a). To determine if the PIN1 inhibitors inhibited the interactions of the NRF2 phospho-peptide mimics with PIN1, FP assays were used in analogous fashion to those with Pintide. First, GST-PIN1 was titrated to the fluorescently labelled NRF2 phospho-peptides (10 nM) in the presence of a fixed concentration of PIN1 inhibitor (100 µM). EGCG did not show any inhibitory effect on the binding of any NRF2 phospho-peptide to PIN1. The presence of juglone led to a 8-, 18-, and sixfold reduction in binding affinity of the fluorescently labelled NRF2_209-222_^pSer215^, NRF2_402-415_^pSer408^, and NRF2_571-584_^pSer577^ NRF2 phospho-peptides respectively (Fig. 6b–d). KPT-6566 was an even more potent inhibitor, inducing a 25-, 92-, and 149-fold reduction in binding affinity of the fluorescently labelled NRF2_209-222_^pSer215^, NRF2_402-415_^pSer408^, and NRF2_571-584_^pSer577^ NRF2 phospho-peptides respectively (Fig. 6b–d). However, none of the curves reached a saturation point meaning the apparent *K*_d_ values obtained are estimated values.

Next, a ligand displacement assay was used to determine if the compounds inhibited binding of the NRF2 peptides to PIN1 in a concentration dependent manner. Thus, the inhibitors were titrated to a fixed concentration of GST-PIN1 (1 µM) and fluorescently labelled NRF2 peptide (10 nM). Juglone inhibited binding in a concentration dependent manner with IC_50_ values of 26.8 µM, 20.0 µM, and 30.0 µM for the NRF2_209-222_^pSer215^, NRF2_402-415_^pSer408^, and NRF2_571-584_^pSer577^ phospho-peptides respectively (Fig. 6e). In alignment with the protein-titration experiments, KPT-6566 showed potent concentration dependent inhibition of PIN1 which was ~ 20-fold more potent than Juglone, with IC_50_ values of 1.3 µM, 0.3 µM, and 1.4 µM for the NRF2_209-222_^pSer215^, NRF2_402-415_^pSer408^, and NRF2_571-584_^pSer577^, respectively (Fig. 6f).

### Mechanism of action for KPT-6566 inhibition of the PIN1- NRF2_209-222_^pSer215^ interaction

**Fig. 7:**
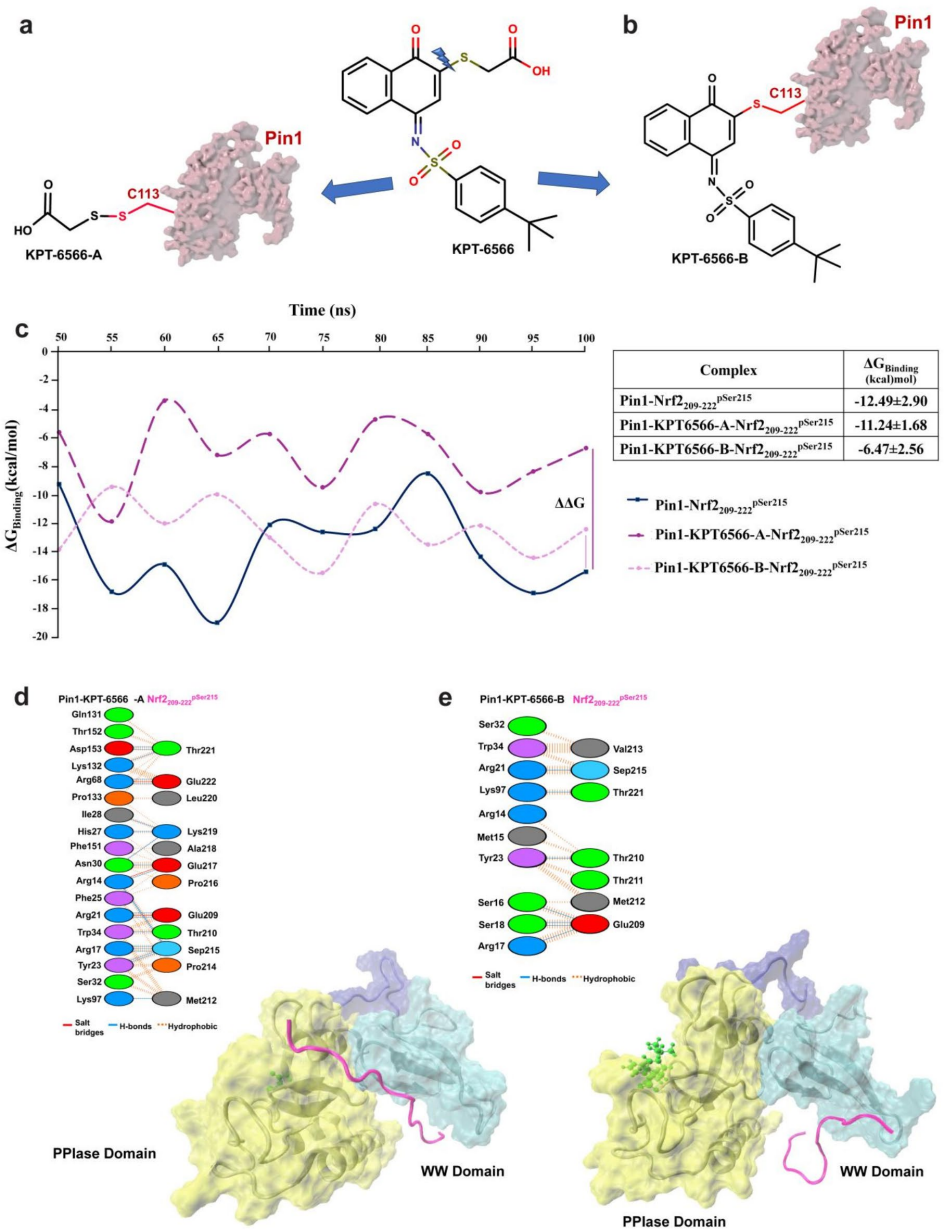
Mechanism of action for KPT-6566 inhibition on PIN1-NRF2 PPI. (**a**,**b**) Transformation of KPT-6566 molecule to PIN1 bound KPT-6566-A and KPT-6566-B molecules. (**c**) Binding energies of PIN1 protein and NRF2_209-222_^pSer215^ peptide obtained from the last 50 ns of MD simulations before and after KPT-6566 modification. (**d**) The binding position of NRF2_209-222_^pSer215^ observed in molecular simulation analyses upon KPT-6566-A modification and diagram showing the interactions between modified PIN1 and NRF2_209-222_^pSer215^ at the amino acid residue level. (**e**) The binding position of NRF2_209-222_^pSer215^ observed in molecular simulation analyses upon KPT-6566-B modification and diagram showing the interactions between modified PIN1 and NRF2_209-222_^pSer215^ at the amino acid residue level.

KPT-6566 was previously shown to covalently modify the PIN1 PPIase domain at Cys113 via an unusual disulfide exchange of the sulfonyl-acetate moiety (KPT-65666-A, Fig. 7a)^31^. This resulted in a PIN1 mass increase of 90 Da in a mass spectrometry experiment^31^. The remaining fragment of KPT-6566 (KPT-6566-B) was reported to be released into the cytoplasm. To verify this, we incubated PIN1 (10 µM) with KPT-6566 (30 µM) and then analysed the results by denaturing mass spectrometry. Under the conditions used, only a small amount of covalent PIN1 modification was observed. Of this, there was only a barely detectable trace of covalent PIN1 modification with KPT-6566-A (Supplementary Fig. 6a, b). The major modified PIN1 species had a mass increase of 353 Da (Supplementary Fig. 6a, c) which corresponded to the product of conjugate addition of KPT-6566 with loss of the sulfonyl-acetate moiety, KPT-6566-B (Fig. 7a,b). Given these findings, the effects of both modes of covalent PIN1 modification by KPT-6566 on NRF2 phospho-peptide binding were subject to computational simulations.

Because the PIN1-NRF2_209-222_^pSer215^ interaction had the lowest simulated relative binding free energy (-12.49 kcal/mol) this peptide sequence was used to simulate the effect of KPT-6566 on NRF2 binding to PIN1.

The relative binding free energy calculations revealed that covalent modification of PIN1 with KPT-6566-A resulted in a slight decrease in the average PIN1-NRF2_209-222_^pSer215^ interaction energy, from − 12.49 kcal/mol to − 11.24 kcal/mol. In contrast, PIN1 modification by KPT-6566-B significantly decreased the interaction energy between PIN1 and NRF2_209-222_^pSer215^ from − 12.49 kcal/mol to − 6.47 kcal/mol (Fig. 7c). This was due to loss of key intermolecular interactions between PIN1 and the peptide (Fig. 7d,e) (Supplementary Table 4,5), possibly due to ligand induced PIN1 allostery. The computational results clearly indicate that the KPT-6566 molecule is expected to have a greater effect when it modifies PIN1 with its KPT-6566-B moiety compared to the sulfonyl-acetate moiety (KPT-6566-A). Together, the computational simulations and mass spectrometry data suggest that modification of PIN1 by KPT-6566-A moiety is not significant in terms of the overall mechanism of action for KPT-6566.

Interestingly, in the study conducted by Campaner et al.^31^, microarray analysis revealed that treatment with KPT-6566 in breast cancer cells resulted in the upregulation of NRF2-mediated oxidative stress elements^31^. Furthermore, quantitative RT-PCR experiments demonstrated the upregulation of NRF2 downstream enzymes, including cFOS, HO1, NQO1, TXNRD1, and DNAJB9, at the mRNA level. This might suggest that KPT-6566 may activate intracellular NRF2 protein somehow. However, there was no direct evidence regarding the effects of KPT-6566 on NRF2 protein and mRNA levels, as well as no evidence related to the compound’s activity on the PIN1-NRF2 PPI specifically. Therefore, additional in vitro cell based assessments of KPT-6566 are necessary to elucidate its effects on the NRF2-PIN1 PPI. In conclusion, while the cytotoxic activity of KPT-6566 against breast cancer cells appears promising, the study by Campaner et al.^31^ highlights a potential concern regarding its NRF2-inducing effect. The upregulation of NRF2-mediated cytoprotective and phase II detoxifying enzymes, without direct evidence of KPT-6566’s effects on NRF2 protein/mRNA levels and its interaction with PIN1, raises questions about its impact on cellular resistance and promotion of a tumour-supportive environment. Further in vitro evaluation is essential to understand the specific effects of KPT-6566 on the PIN1-NRF2 PPIs and its potential implications for cancer therapy.

## Discussion

The phosphorylation dependent interaction of NRF2 with PIN1 underpins a mechanism of NRF2 regulation that is poorly understood. The NRF2 residues essential for this PPI (Ser215, Ser408, Ser577) have been identified via cell-based approaches^16^, but the molecular basis for the interaction has not previously been investigated. Here, we present the results of an integrated computational, biophysical and biochemical study that provides the first insight into this interaction at the molecular level.

In the current study, three different phospho-peptides were used to mimic NRF2 binding sites: NRF2_209-222_^pSer215^, NRF2_402-415_^pSer408^, and NRF2_571-584_^pSer577^. FP experiments revealed that the three phospho-peptides bound to PIN1 with comparable apparent Kds (157, 221, 168 nM respectively) which are significantly lower than for other PIN1 peptide substrates mimicking native binding partners like Tau which fall in the micro-molar range^32^. This is an indication that these are physiologically relevant binding affinities, however, they are tenfold weaker than Pintide, a synthetic phospho-peptide optimised for binding to the PIN1 WW domain. Computational simulations indicated that all three peptides interacted with the PIN1 WW domain, but had different binding free energies, with NRF2_209-222_^pSer215^ having the most favourable. The simulations identified a common PIN1 WW domain binding site involving Ser 16, Arg 17, Ser 18, Tyr 23, Ser 32, Gln 33, and Trp 34, which is consistent with previous studies on PIN1 substrate binding. Further confirmation of the binding site was obtained via FP competition assays with the well characterised Pintide ligand. These results provide confirmation that, upon phosphorylation, the NRF2 regions around Ser215, Ser408 and Ser577 are high affinity recognition motifs for PIN1 binding. It is not yet clear if these sites are also substrates for PIN1-induced proline isomerisation. PIN1 is known to recognise multiple pSP sites within the same protein substrate which might be an important mechanistic requirement for transfer of substrate from the high affinity WW-domain to the lower affinity PPIase domain^33,34^. Given the multitude of proteins known to interact with PIN1 it is also possible that PIN1 regulates NRF2 activity via direct PPIs alone. As discussed previously, the PIN1 binding motifs on the NRF2 protein are positioned near the binding sites of NRF2 negative regulator proteins such as RXRα, RARα, β-TrCP, and CHD6. Consequently, PIN1 stabilisation of NRF2 may result from inhibition of negative regulator binding.

The effects of small molecule PIN1 inhibitors on the NRF2-PIN1 interaction were also investigated. EGCG, a compound reported to interact with both the WW domain and catalytic domain of PIN1^35^, did not show any effect. The reported covalent PIN1 inhibitors juglone and KPT-6566 did impede NRF2 peptide binding, with KPT-6566 being the most potent with IC_50_ values of 1.3, 0.3, and 1.4 µM for NRF2_209-222_^pSer215^, NRF2_402-415_^pSer408^, and NRF2_571-584_^pSer577^ respectively. LC–MS analysis indicated that PIN1 was covalently modified by KPT-6566 to a small degree. Contrary to a previous report, in our study KPT-6566 appeared to covalently modify PIN1 via conjugate addition, rather than disulfide exchange of the sulfonyl-acetate moiety^31^. The precise location of this modification was not confirmed, but based on previous studies it is presumed to be Cys113 in the PIN1 PPIase domain. Moreover, it should also be emphasized that there is no experimental validation for parametrization of covalently bound ligands for MD simulations. The method employed in the study was validated with experimental results for a non-covalent ligand^36^. However, the consistency in the result of multi-repeated simulations and consistency between computational and experimental results validate the use of this parameterization method. The detailed explanation and studies validating it can be found in Supplementary Information File. Computational simulations predicted that both covalent modifications would increase the free binding energy of the PIN1 interaction with NRF2_209-222_^pSer215^, but that modification of PIN1 with KPT-6566-B via conjugate addition would have a much greater inhibitory effect. The fact that a small-molecule ligands assumed to bind to PIN1 in its PPIase domain inhibits the binding of recognition motifs to the WW domain is intriguing. This could be a result of dynamic allosteric interplay between the PIN1 PPIase and WW domains which has been widely reported^37,38^. Alternatively, juglone and KPT-6566 might act via two distinct mechanisms simultaneously – a covalent modification mechanism, and an allosteric non-covalent mechanism. In both cases harnessing and controlling these effects using small molecules in a specific manner could underpin novel drug design strategies. The development of such new-generation small molecule PIN1 inhibitors that mitigate the effects of PIN1 overexpression in cancer cells could reignite PIN1 drug development efforts. Such inhibitors would also be highly valuable molecular probes for further investigation of PIN1 regulation of NRF2 in the cellular context, and potentially pave the way for drug molecules that specifically inhibit the cytoprotective effects of NRF2 in cancer.

## Methods

### Preparation of bacterial stab culture, protein expression, and purification

The GST-PIN1 (#19027, Addgene) plasmid construct was obtained from commercial bacterial stab cultures. Colonies from these cultures were inoculated in 5 mL of terrific broth medium and grown at 37 °C with vigorous shaking for 18 h to yield sufficient bacterial culture for DNA isolation. Plasmid DNA was isolated using the E.Z.N.A.® Plasmid DNA Mini Kit I (#D6942-01, omega BIO-TEK) with slight modifications. The pelleted bacterial culture was washed sequentially with provided buffers and subjected to centrifugation processes. The membrane-bound DNA was then eluted with 70 µL of elution buffer and stored at − 20 °C. The quality and concentration of plasmid DNA was assessed by NanoDrop™ 2000 spectrophotometer and DNA sequencing.

Three microliters of purified plasmids (~ 50 ng/µL) were transfected to 50 µL of BL21 (DE3) competent cells by heat shock transformation and plated on standard LB agar. Positive BL21 (DE3) clones were selected with ampicillin treatment. A single colony was inoculated in ampicillin-containing (100 μg/mL) terrific broth and grown at 37 °C to an optical density of 0.7. Gene expression was induced with 0.4 mM of isopropyl β-D-1-thiogalactopyranoside (IPTG) for 18 h at 25 °C. The grown cells were collected by centrifugation at 5,000 rpm for 20 min at 4 °C. For GST-PIN1 protein isolations, cell pellets were suspended in 15 mL of lysis buffer (50 mM HEPES, 300 mM NaCl, 1.0 mM DTT, and pH 7.4). The PIN1 proteins with GST tags were purified from the total protein lysates by using Glutathione spin columns (#16105, Thermo Scientific Pierce™) equilibrated with lysis buffer.

### Fluorescence polarisation (FP) assay

Fluorescence polarisation experiments were performed at room temperature in a buffer consisting of 5 mM HEPES pH 7.4, 30 mM NaCl, 0.1% v/v Tween20, and 1% v/v DMSO. Corning black, round-bottom, low-binding 384-well plates were utilized for all assays. The NRF2 mimicking fluorescein-labelled peptides, ETTMVPpSPEAKLTE (NRF2_209-222_^pSer215^), DMVQPLpSPSQGQST (NRF2_402-415_^pSer408^), and EDGKPYpSPSEYDLQ (NRF2_571-584_^pSer577^) and the Pintide peptide (WFYpSPFLE) with the optimal binding for the WW domain were commercially purchased from China Peptides. For protein titration studies, GST-PIN1 was titrated to the fluorescently-labelled Nrf 2 phospho-peptides (10 nM) in the presence of a fixed concentration of PIN1 inhibitor (100 μM) or only DMSO as control group. To show PIN1 specificity, only GST protein was used as the control protein group. Fluorescence polarisation was measured using a CLARIOstar® Microplate Reader with an excitation wavelength of λex: 490/20 nm and an emission wavelength of λem: 535/20 nm. The binding affinities (*K*_d_) were determined by sing GraphPad Prism 7 software, and sigmoidal curves were fitted using the equation: Y = Bottom + (Top − Bottom)/(1 + 10^((Log app.Kd − X) * HillSlope)), where Y represents the mP value, X denotes the log protein concentration, and Top and Bottom represent the plateaus in mP. Moreover, for the ligand displacement assays, the inhibitors were titrated to a fixed concentration of GST-PIN1 (1 μM) and fluorescently labelled NRF2 peptide (10 nM). The change in polarisation values were normalised to the DMSO control group and presented as percentage change.

## Mass spectrometry analyses

From protein stock, PIN1 was diluted into an assay buffer solution of 50 mM HEPES, 300 mM NaCl pH 7.4 to a final concentration 10 μM and incubated with KPT-6566 (30 μM) for 1 h. Then, the sample was 2-times diluted by using H_2_O containing 0.1% v/v formic acid solution to give a final protein concentration of 0.1–1 mg/mL, and mass spectra were obtained using a Waters Acquity XEVO Q ToF instrument.

### In silico analyses

PIN1 amino acids interacting with NRF2 mimicking peptides were elucidated through molecular docking and molecular dynamics simulations. These simulations were followed by binding energy calculations the stronger binding peptide was determined. Lastly, the comparison of binding energies for PIN1-NRF2_209-222_^pSer215^ and KPT6566 bound PIN1-NRF2_209-222_^pSer215^ 1 complexes were performed.

### Modelling of PIN1 protein and NRF2 mimicking peptides

In the experimentally revealed structures the linker region (Gly 43-Gln 53) was missing due to increased flexibility; thus, the complete predicted structure from the Alphafold database was selected (ID: AF-Q13526-F1) and downloaded as the starting PIN1 structure. The first residue, methionine (Met 1), in the structure was mutated to asparagine (Asn 1) utilising YASARA Structure software^39^ to ensure consistency with the identical PIN1 sequence employed in the FP assay.

The NRF2 mimicking peptides were modelled by using two online servers. Initially, non-phosphorylated forms of the peptides were designed in the PEPstrMOD server^40,41^ with the following sequences; ETTMVPpSPEAKLTE (NRF2_209-222_^pSer215^), DMVQPLpSPSQGQST (NRF2_402-415_^pSer408^), and EDGKPYpSPSEYDLQ (NRF2_571-584_^pSer577^). The phosphorylated serine residues are pivotal for the interaction between NRF2 and PIN1^16^. Thus, serine residues at the 7^th^ position of all three designed peptides were modelled as phosphorylated amino acids. Phosphate groups with 2- charges were added from the hydroxyl groups of serine residue side chains utilising the Vienna-PTM online server^42–44^.

### Molecular dynamics (MD) simulations

All MD simulations in the study were performed with YASARA Structure software v.21.12.19^39^. The setup involved optimising the hydrogen bonding network to enhance solute stability and predicting p*K*_*a*_ values to refine the protonation states of protein residues at a selected pH of 7.4^45^. Titratable amino acid side chains were protonated according to these p*K*_*a*_ calculations. Then each protein was placed into a cubic box with the specific dimensions as written explicitly below. The cell was filled with water molecules. TIP3P water model was utilised, whose density was set at 0.997 g/ml at temperature of 300 K. Then, NaCl ions were added with a physiological concentration of 0.9% with an excess of either Na^+^ or Cl^-^ to neutralise the cell. Force field for all simulations was AMBER 14^46^. The cutoff was 8 Å for Van der Waals forces (the default used by AMBER^47^), no cutoff was applied to electrostatic forces (using the Particle Mesh Ewald algorithm^48^). The equations of motions were integrated with 2.5 femtoseconds (fs) timestep for bonded interactions and 5.0 fs for non-bonded interactions at 300 K and a pressure of 1 atm (NPT ensemble) using algorithms described in detail previously^45^. After steepest descent and simulated annealing minimizations were run to remove clashes, and finally the production simulations were performed.

PIN1 protein was placed into a cubic box with dimensions 6.72 × 6.72 × 6.72 nm and the production simulations were run for 200 ns (ns). After analysing the RMSD vs simulation time graphs for PIN1 protein the last 50 ns was considered as equilibrated periods, and cluster structure was obtained from this period.

The 3-D structures of the designed peptides; ETTMVPpSPEAKLTE (NRF2_209-222_^pSer215^), DMVQPLpSPSQGQST (NRF2_402-415_^pSer408^), and EDGKPYpSPSEYDLQ (NRF2_571-584_^pSer577^) were designed at PEPstrMOD server^40,41^. The designed peptides were simulated at pH = 7.4 and 300 K conditions. NRF2_209-222_^pSer215^ was placed into a cubic box of dimensions 3.5 × 3.5 × 3.5 nm, NRF2_402-415_^pSer408^ was placed into a cubic box with dimensions of 4.8 × 4.8 × 4.8 nm, and NRF2_571-584_^pSer577^ was placed into a cubic box with dimensions 3.5 × 3.5 × 3.5 nm. All the parameters and the steps involving MD simulations were the same as explained in detail above. Each of the peptides was subjected to 100 ns-long classical MD simulations. After analysing the RMSD vs simulation time graphs for peptides, for NRF2_209-222_^pSer215^ the last 20 ns period of the simulation, for NRF2_402-415_^pSer408^ the last 15 ns period, and for NRF2_571-584_^pSer577^ the last 15 ns period were considered as equilibrated periods, and cluster structures were obtained from those periods. The cluster structures of peptides were docked to the PIN1 protein cluster structure in the next step.

### PIN1-peptides complexes

PIN1-peptide complexes were obtained from protein–protein docking simulations, whose details were described below. Each PIN1-peptide complex was placed into a cubic box with dimensions of 7.0 × 7.0 × 7.0 nm (PIN1- NRF2_209-222_^pSer215^), 7.0 × 7.0 × 7.0 nm (PIN1- NRF2_402-415_^pSer408^), and 7.5 × 7.0 × 8.0 nm (PIN1- NRF2_571-584_^pSer577^) and the production simulations were run for 100 ns. After analysing the RMSD vs simulation time graphs for each PIN1-peptide complex, the last 30 ns were considered as equilibrated periods, and cluster structures were obtained from these periods. The snapshots of complexes were obtained from the last 50 ns for relative binding free energy calculations. These relative binding free energy calculations were conducted using FoldX, which calculates relative binding free energies (ΔG_binding​)_) by combining multiple energy terms derived from both experimental and empirical data. The parameters were optimized for PPIs, including a temperature of 298 K and an ionic strength of 0.05 M. The analysis involved energy decomposition and the calculation of the Gibbs free energy of binding to evaluate the interaction between PIN1 and the phosphopeptides^49^. The mathematical formalism and detailed equations used in these calculations are provided in the Supplementary Information File.

### Covalent KPT-PIN1- NRF2_209-222_^pSer215^ complexes

MD simulations of KPT-6566-A and KPT-6566-B bound to PIN1 protein using the conditions detailed above were placed into the same boxes of dimensions 70 × 70 × 70 Å and subjected to 100 ns molecular dynamics simulations. Ligands were parameterized with AutoSMILES protocol implemented in YASARA Structure, using GAFF2^50^ and AM1BCC^51^. AutoSMILES is a highly automated and advanced tool for parameter assignment that utilizes SMILES strings to identify known molecules, even when residue and atom names differ. For unrecognized molecules, AutoSMILES applies the General AMBER Force Field (GAFF) and AM1-BCC charge assignment methods to ensure accurate representation. Additionally, AM1-BCC charges are refined using known RESP charges of structurally similar molecular fragments, also identified via SMILES strings. This approach ensures a robust parameterization process for complex molecules, such as KPT-6566, and integrates seamlessly into the simulation workflow. After analysing the RMSD vs simulation time graphs for PIN1-KPT-6566-A and PIN1-KPT-6566-B complexes, the last 20 ns were considered as equilibrated periods, and cluster structures were obtained from these periods. Peptide 1 cluster structure was docked to the cluster structures of PIN1-KPT-6566-A and PIN1-KPT-6566-B in the next step.

### Protein-peptide molecular docking

All protein-peptide molecular docking simulations were performed at HPEPDOCK 2.0 online server^52–57^. The most representative 3D structures of the PIN1 or KPT-PIN1 complexes were uploaded into the server as a receptor molecule. In the HPEPDOCK 2.0 server, SEP is the recommended entity used to represent phosphorylated serine residues within proteins or peptides. Therefore, before submitting the peptides to the server, the phosphorylated serine residues within their structures were specifically identified as SEP. Molecular docking analyses were performed with default parameters. Interactions between protein-peptide complexes were visualised in YASARA Structure software^39^.

### Covalent ligand molecular docking

Based on the mass spectrometry data, in our study, KPT-6566 was cleaved into two parts as KPT-6566-A and KPT-6566-B (Fig. 7a). These two subunits were first sketched on Marvin 15.12.14 software^58^. Geometry optimization of KPT-6566 was performed using YASARA Structure software with NOVA force field^59^.

As it is known, KPT-6566 binds to a cysteine C113 residue of the PIN1 catalytic core. Therefore, KPT-6566-A and KPT-6566-B were covalently docked to C113 residue of PIN1. Covalent docking simulations were performed on YASARA Structure software (v21.12.19, YASARA Biosciences GmBH, Wien, Austria), which utilised AutoDock software^60^. AutoDock considers the covalently bound ligand as a flexible side chain^60^. Each docking trial yielded 25 poses with an exhaustiveness value of 25, with slight variations each. The pose with the highest binding affinity was considered the best and this top pose was discussed.

## Electronic supplementary material

Below is the link to the electronic supplementary material.

Supplementary Material 1

Supplementary Material 2

## Data Availability

The authors declare that all data generated or examined throughout this research has been included in this published article and its supplementary information files. The PDB structures used in this study is available in the AlphaFold Protein Structure Database (ID: AF-Q13526-F1). Additional datasets used and/or analyzed during the current study are available upon reasonable request from the corresponding authors T.B.T., and R.G.D.
