# Supplementary material for: Identification and inhibition of PIN1-NRF2 protein–protein interactions through computational and biophysical approaches
Source: Sci Rep. 2025 Mar 14;15:8907. doi: 10.1038/s41598-025-89342-0 (PMC11909128; doi:10.1038/s41598-025-89342-0)
Supplement: Supplementary file 1 — Supplementary Material 1 [file 41598_2025_89342_MOESM1_ESM.docx]

**Identification and Inhibition of PIN1-NRF2 Protein-Protein Interactions through Computational and Biophysical Approaches**

Adem Ozleyen^1,2,3^, Gizem Nur Duran^4^, Serhat Donmez^5,6^*,* Mehmet Ozbil^4^, Richard G Doveston^1,2,*^, Tugba Boyunegmez Tumer^7,8*^

*^1^ Leicester Institute for Structural and Chemical Biology, University of Leicester, Leicester, LE1 7RH United Kingdom*

*^2^* *School of Chemistry, University of Leicester, Leicester, LE1 7RH United Kingdom*

*^3^ Health Institutes of Türkiye, Türkiye Biotechnology Institute, Ankara, 06270 Türkiye*

*^4^ Institute of Biotechnology, Gebze Technical University, Gebze, Kocaeli, 41400 Türkiye*

*^5^* *Graduate Program of Molecular Biology and Genetics, School of Graduate Studies, Canakkale Onsekiz Mart University, Canakkale, 17020 Türkiye*

*^6^ Institute of Science and Technology Austria (ISTA), Klosterneuburg, 3400 Austria*

*^7^ Department of Molecular Biology and Genetics, Faculty of Arts and Science, Canakkale Onsekiz Mart University, Canakkale, 17020 Türkiye*

*^8^Department of Medical Biotechnology, Faculty of Biochemistry, Biophysics and Biotechnology, Jagiellonian University, Krakow, Poland*

***Author Information***

***Corresponding Authors***

**E-mails: tumertb@comu.edu.tr, tugba.tumer-styp@uj.edu.pl (TBT) and r.g.doveston@leicester.ac.uk (RGD)*


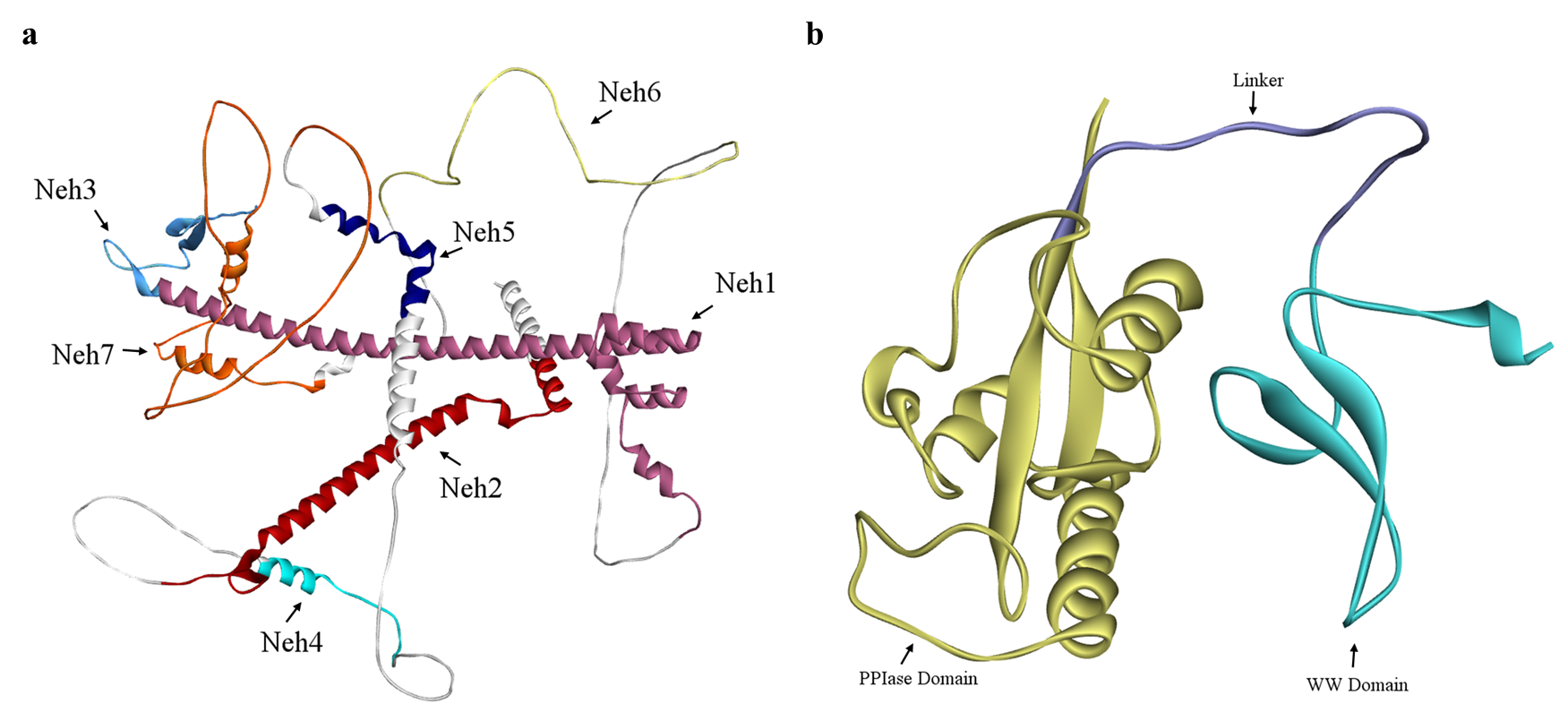


**Supplementary Fig. 1: 3D structure of human NRF2 and PIN1.** The complete predicted structure of (a) NRF2 and (b) PIN1 were derived from the Alphafold database (NRF2 ID: AF-Q13526-F1 and PIN1 ID:AF-Q16236-F1).

**
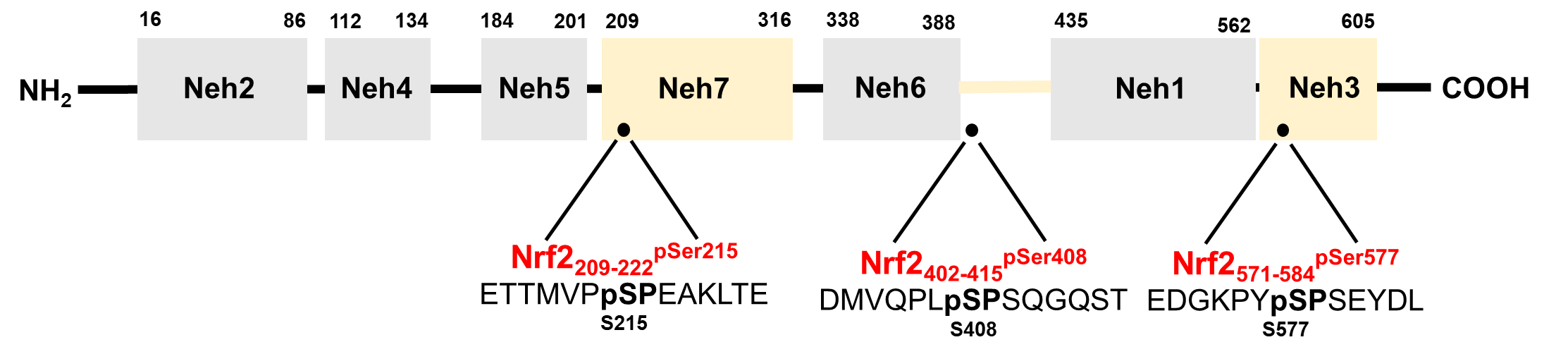
**

**Supplementary Fig. 2: The binding sites of NRF2-PIN1 PPIs.** For the computational calculations and FP analyses, 3 different 14-mer long phosphorylated peptides mimicking NRF2 protein (NRF2_209-222_^pSer215^, NRF2_402-415_^pSer408^, and NRF2_571-584_^pSer577^) were used.


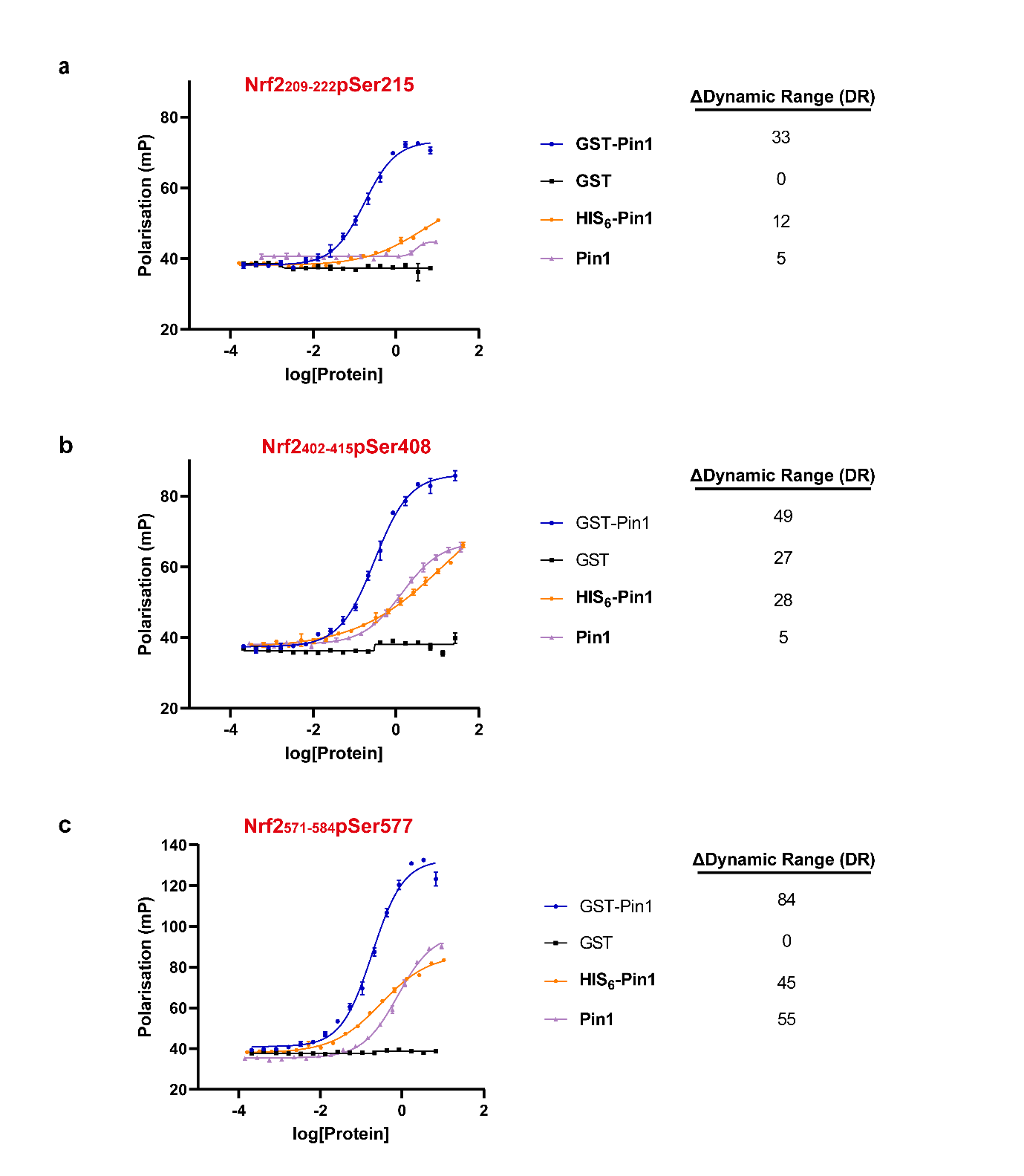


**Supplementary Fig. 3: Determination of PIN1 protein construct for performing FP assay.** GST-PIN1, HIS_6_-PIN1, and PIN1 were titrated to fluorescently labelled (a) NRF2_209-222_^pSer215^, (b) NRF2_402-415_^pSer408^, and (c) NRF2_571-584_^pSer577^ peptides (10 nM), separately. GST protein was used as a control (black line). Error bars represent standard deviation for n=3 replicates. Tables next to each graphs summarise the dynamic ranges of obtained curves.


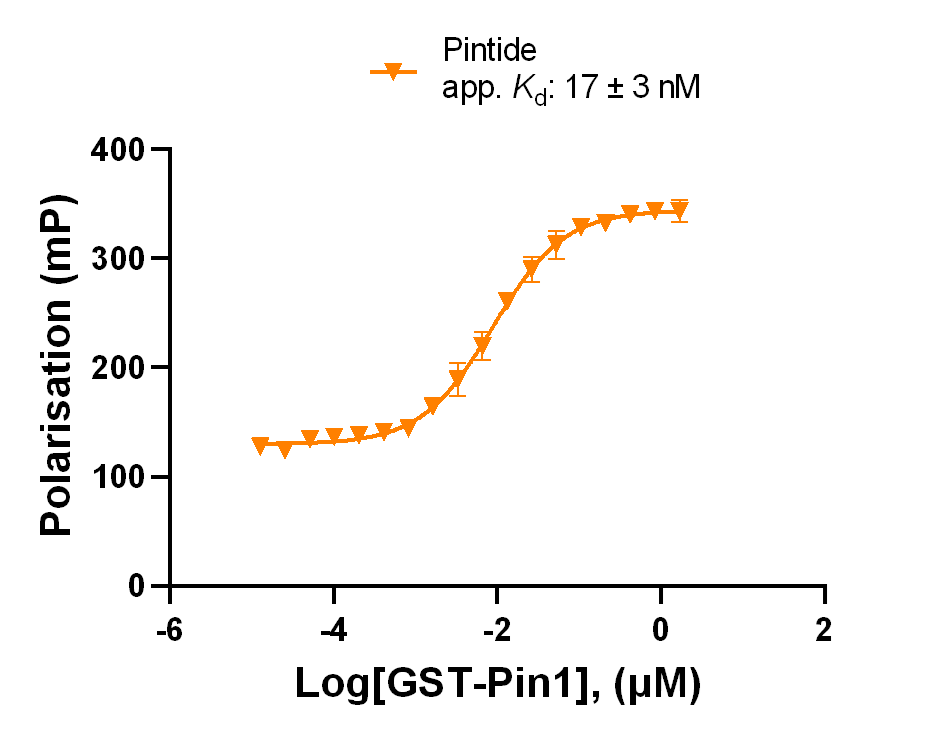


**Supplementary Fig. 4: The binding affinity of Pintide peptide.** FP data to assess the binding affinity of pintide with GST-PIN1. GST-PIN1 was titrated to fluorescently labelled pintide peptide (10 nM). Error bars represent standard deviation for n=3 replicates.


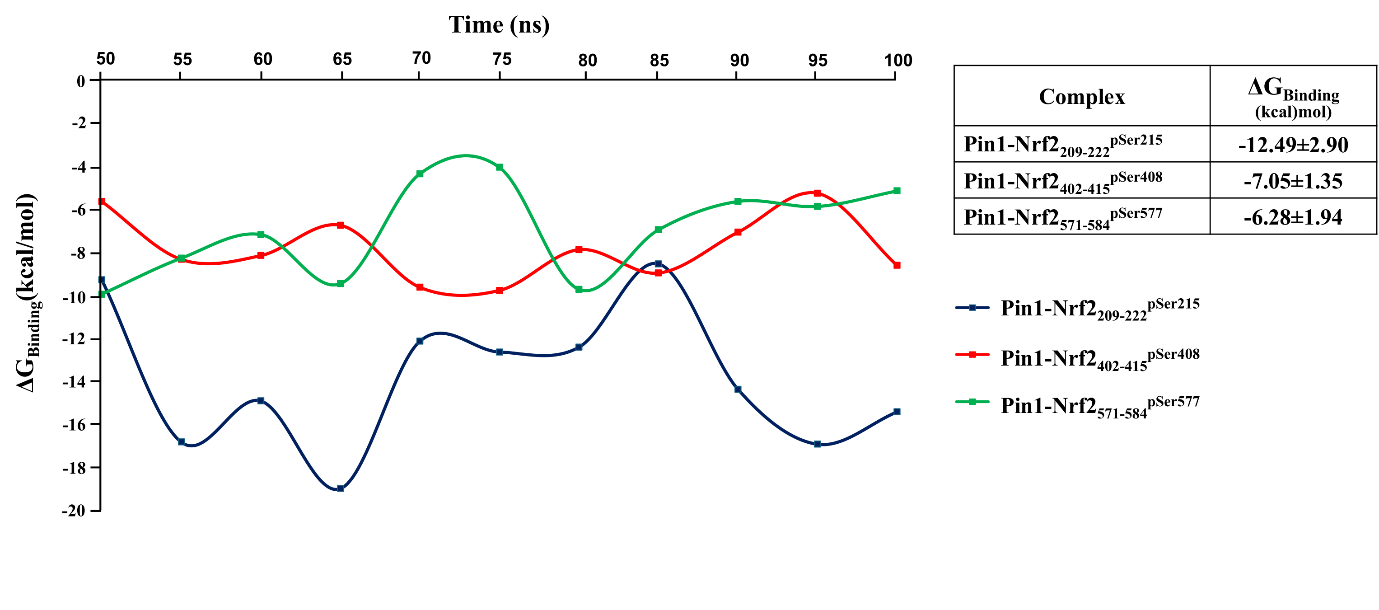


**Supplementary Fig. 5:** Binding energies of PIN1 protein and NRF2_209-222_^pSer215^, NRF2_402-415_^pSer408^, and NRF2_571-584_^pSer577^ peptides obtained from the last 50 ns of MD simulations. The average binding energies with their standard deviations for provided in the table.


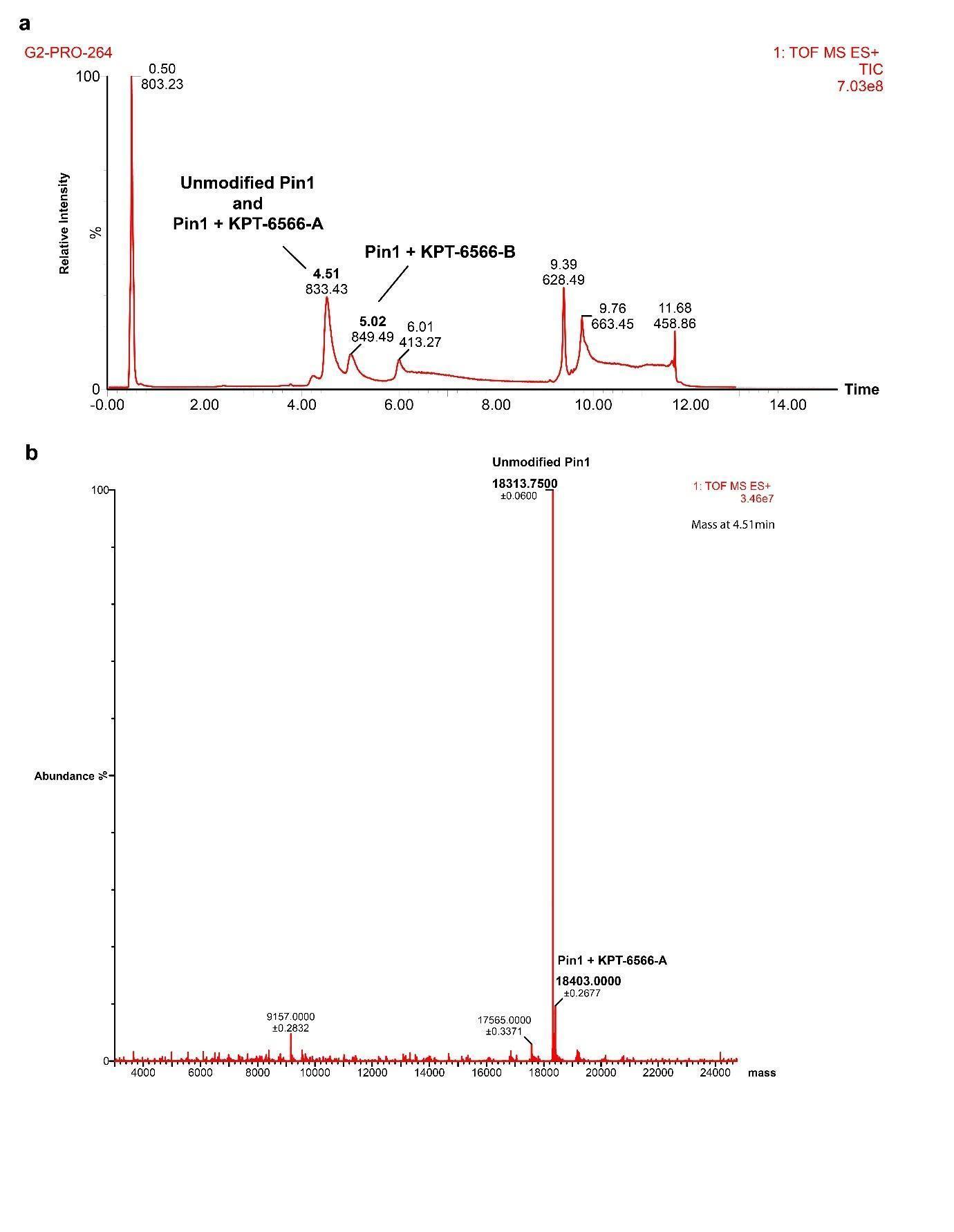


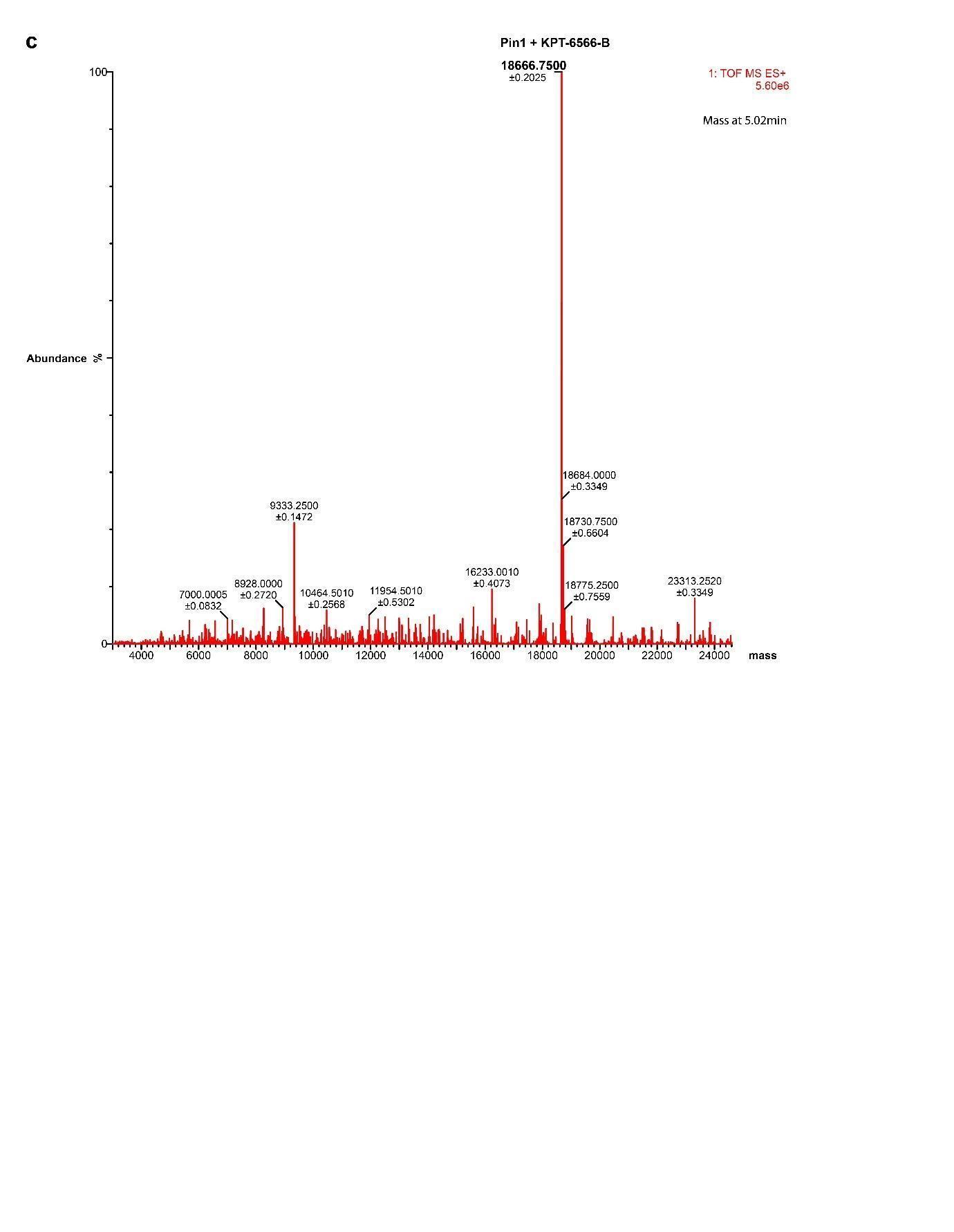


**Supplementary Fig. 6: Mass spectrometry data showing PIN1 modification with KPT-6566.** (a) The spectra represent relative intensity against time. (b) Denaturing mass spectrometry showing abundance of unmodified PIN1 and PIN1 modified with KPT-6566-A. (c) Denaturing mass spectrometry showing abundance of unmodified PIN1 and PIN1 modified with KPT-6566-B.

**Supplementary Table 1.** Intermolecular non-covalent interactions between NRF2_209-222_^pSer215^ and PIN1.

| **PIN1 - NRF2_209-222_^pSer215^** | |
| --- | --- |
| **Interacting Amino Acids** | **Interaction Types** |
| Asn 1 | H-Bond / Hydrophobic |
| Ala 2 | H-Bond / Hydrophobic |
| Asp 3 | H-Bond / Hydrophobic |
| Glu 4 | H-Bond / Hydrophobic |
| Lys 13 | Hydrophobic |
| Arg 14 | H-Bond / Hydrophobic |
| Met 15 | H-Bond / Hydrophobic |
| Ser 16 | H-Bond / Hydrophobic |
| Arg 17 | H-Bond / Hydrophobic |
| Ser 18 | H-Bond / Hydrophobic |
| Arg 21 | H-Bond / Hydrophobic / Salt Bridge |
| Tyr 23 | H-Bond / Hydrophobic |
| Phe 25 | Hydrophobic |
| Asn 30 | Hydrophobic |
| Ser 32 | H-Bond / Hydrophobic |
| Gln 33 | Hydrophobic |
| Trp 34 | H-Bond / Hydrophobic |
| Lys 82 | H-Bond / Hydrophobic / Salt Bridge |
| Leu 86 | Hydrophobic |
| Glu 87 | H-Bond / Hydrophobic / Salt Bridge |
| Asn 90 | H-Bond / Hydrophobic |
| Gln 94 | Hydrophobic |
| Lys 97 | H-Bond / Hydrophobic |
| Pro 149 | Hydrophobic |
| Phe 151 | Hydrophobic |

**Supplementary Table 2.** Intermolecular non-covalent interactions between NRF2_402-415_^pSer408^ and PIN1.

| **PIN1 - NRF2_402-415_^pSer408^** | |
| --- | --- |
| **Interacting Amino Acids** | **Interaction Types** |
| Glu 12 | Hydrophobic |
| Lys 13 | H-Bond / Hydrophobic |
| Arg 14 | H-Bond / Hydrophobic |
| Met 15 | H-Bond / Hydrophobic |
| Ser 16 | H-Bond / Hydrophobic |
| Arg 17 | H-Bond / Hydrophobic |
| Ser 18 | H-Bond / Hydrophobic |
| Ser 19 | H-Bond / Hydrophobic |
| Arg 21 | H-Bond / Hydrophobic |
| Tyr 23 | H-Bond / Hydrophobic |
| Ser 32 | Hydrophobic |
| Gln 33 | Hydrophobic |
| Trp 34 | H-Bond / Hydrophobic |

**Supplementary Table 3.** Intermolecular non-covalent interactions between NRF2_571-584_^pSer577^ and PIN1.

| **Pin 1 – NRF2_571-584_^pSer577^** | |
| --- | --- |
| **Interacting Amino Acids** | **Interaction Types** |
| Ser 16 | H-Bond / Hydrophobic |
| Arg 17 | H-Bond / Hydrophobic |
| Ser 18 | Hydrophobic |
| Tyr 23 | H-Bond / Hydrophobic |
| Asn 30 | Hydrophobic |
| Ser 32 | H-Bond / Hydrophobic |
| Gln 33 | Hydrophobic |
| Trp 34 | H-Bond / Hydrophobic |
| Arg 36 | Hydrophobic |
| Pro 37 | Hydrophobic |
| Ser 38 | H-Bond / Hydrophobic |
| Ile 93 | Hydrophobic |
| Gln 94 | H-Bond |
| Ile 96 | Hydrophobic |
| Lys 97 | H-Bond / Hydrophobic |
| Arg 142 | H-Bond / Hydrophobic / Salt Bridge |
| Gly 148 | Hydrophobic |
| Pro 149 | Hydrophobic |

**Supplementary Table 4.** Intermolecular non-covalent interactions between NRF2_209-222_^pSer215^ and KPT-6566-A-modified PIN1.

| **PIN1-KPT-6566-A-NRF2_209-222_^pSer215^** | |
| --- | --- |
| **Interacting Amino Acids** | **Interaction Types** |
| Arg 14 | H-Bond / Hydrophobic / Salt Bridge |
| Arg 17 | H-Bond / Hydrophobic |
| Arg 21 | H-Bond / Hydrophobic / Salt Bridge |
| Tyr 23 | H-Bond / Hydrophobic |
| Phe 25 | Hydrophobic |
| His 27 | H-Bond / Hydrophobic |
| Ile 28 | H-Bond / Hydrophobic |
| Asn 30 | H-Bond / Hydrophobic |
| Ser 32 | Hydrophobic |
| Trp 34 | H-Bond / Hydrophobic |
| Arg 68 | H-Bond / Hydrophobic / Salt Bridge |
| Lys 97 | H-Bond / Hydrophobic |
| Gln 131 | Hydrophobic |
| Lys 132 | Hydrophobic |
| Pro 133 | Hydrophobic |
| Phe 151 | Hydrophobic |
| Thr 152 | Hydrophobic |
| Asp 153 | Hydrophobic |

**Supplementary Table 5.** Intermolecular non-covalent interactions between NRF2_209-222_^pSer215^ and KPT-6566-B-modified PIN1.

| **PIN1-KPT-6566-B-NRF2_209-222_^pSer215^** | |
| --- | --- |
| **Interacting Amino Acids** | **Interaction Types** |
| Arg 14 | Hydrophobic |
| Met 15 | Hydrophobic |
| Ser 16 | H-Bond / Hydrophobic |
| Arg 17 | H-Bond / Hydrophobic |
| Ser 18 | H-Bond / Hydrophobic |
| Arg 21 | H-Bond / Hydrophobic |
| Tyr 23 | H-Bond / Hydrophobic |
| Ser 32 | Hydrophobic |
| Trp 34 | Hydrophobic |
| Lys 97 | H-Bond / Hydrophobic |
