# Supplementary material for: Identification and inhibition of PIN1-NRF2 protein–protein interactions through computational and biophysical approaches
Source: Sci Rep. 2025 Mar 14;15:8907. doi: 10.1038/s41598-025-89342-0 (PMC11909128; doi:10.1038/s41598-025-89342-0)
Supplement: Supplementary file 2 — Supplementary Material 2 [file 41598_2025_89342_MOESM2_ESM.docx]

The relative binding free energy values calculated with FoldX have been used to understand the ranking of binding strengths between peptides and Pin1. FoldX is calibrated to predict energy differences (relative values) with a high degree of correlation to experimental data (R² = 0.81). However, its absolute free energy values are subject to approximations inherent in the empirical approach and should not be considered precise. In our study, the ranking of peptides' relative binding free energies (e.g., Nrf_209−222_pSer215 having the strongest binding) aligned well with experimental data from fluorescence polarization (FP) assays^1^.

FoldX calculates the relative binding free energies (ΔG_binding​)_ by combining multiple energy terms derived from both experimental and empirical data, as detailed in its formalism^1^:

$\Delta G=a.{\Delta G}_{vdw}+b.{\Delta G}_{solvH}+c.{\Delta G}_{solvP}+{d.\Delta G}_{wb}+e.{\Delta G}_{hbond}+f.{\Delta G}_{el}+g.{\Delta G}_{kon}+h.{T\Delta S}_{mc}+k.{T\Delta S}_{sc}+l.{\Delta G}_{clash}$

Here are the main contributions:

1. **Van der Waals Interactions (**${\Delta G}_{vdw}$**):**Based on atom proximity and experimental transfer energies between water and vapor.
2. **Solvation Energy (**${\Delta G}_{solvH} and {\Delta G}_{solvP}$**):**

- ${\Delta G}_{solvH}$**_:_** Contribution from hydrophobic groups during desolvation.
- ${\Delta G}_{solvP}$**:** Contribution from polar groups during desolvation.
  Solvation parameters are derived from experimental transfer of amino acids from water to organic solvents.

1. **Bound Water Molecules (**${\Delta G}_{wb}$**):**
   Represents the energetic contribution of water molecules that persistently interact with the protein by forming more than two hydrogen bonds.
2. **Hydrogen Bonds (**${\Delta G}_{hbond}$**):**Calculated based on geometric criteria and inferred from protein engineering double mutant cycles.
3. **Electrostatic Interactions (**${\Delta G}_{el}$**):**
   Computed using Coulomb's law with the dielectric constant scaled by the burial of the interaction.
   Includes additional virtual atoms to capture specific effects such as helix dipoles and aromatic ring charges.
4. **Protein-Protein Complex Interactions (**${\Delta G}_{kon}$**):**Accounts for electrostatic contributions between polypeptide chains, estimated using an empirical equation that predicts the association rate ($G_{kon}$​).
5. **Entropy Contributions:**

- **Main-chain entropy (**${T\Delta S}_{mc}$**​):** Derived from the statistical analysis of phi-psi distributions observed in crystal structures and scaled by accessibility and hydrogen bonding energetics.
- **Side-chain entropy (**${T\Delta S}_{sc}$**):** Calculated based on predefined entropy parameters scaled by side-chain burial.

1. **Steric Clashes (**${\Delta G}_{clash}$**):**
   Quantifies steric overlaps between atoms.

- Soft penalization is recommended for point mutation analysis.
- Full penalization is applied for protein design.

The FoldX approach explicitly incorporates entropy through terms that model the loss of conformational freedom for backbone and side chains upon complex formation. This inclusion is particularly relevant when estimating relative binding free energies.

In our study, we employed YASARA’s AutoSMILES protocol to parameterize the covalent ligand KPT-6566. The parameters for the ligand were generated using AutoSMILES. AutoSMILES streamlines parameterization by integrating SMILES-based molecule identification with advanced charge and force field assignment techniques. Replacing RESP charges with AM1-BCC charges in the Cornell et al.^2^ force field produced a model capable of accurately reproducing correlated ab initio energies for hydrogen-bonded organic homo- and hetero-dimers, with an average deviation of 0.95 kcal/mol, and for hydrogen-bonded DNA dimers, with an average deviation of 0.9 kcal/mol. The AM1-BCC charge model demonstrated a consistently high correlation coefficient exceeding 0.96 in all validation tests, aligning closely with both ab initio calculations and experimental data. As a result, this approach is proposed as a reliable and general method for assigning charges to organic molecules in solution phase simulations^3^. It should be emphasized that there is no experimental validation of this method for covalently bound ligands. However, the consistency in the result of multi-repeated simulations and consistency between computational and experimental results validate the use of this parameterization method.

The protocol begins by assigning pH-dependent protonation states and fractional bond orders to ensure compatibility with physiological conditions^4^. Known molecules are directly matched to force field parameters, while novel molecules, like KPT-6566, undergo further parameterization^5^.

For uncharacterized molecules, AM1 Mulliken charges are calculated following geometry optimization in the COSMO solvation model, preventing artifacts in highly charged species^6^. AM1-BCC charges are then assigned and refined by comparing to known RESP charges of similar molecular fragments identified via SMILES strings^7^. Remaining parameters, such as atom types, are assigned using the GAFF force field, optimized for small organic molecules^5^.

AutoSMILES generates a complete parameter set, including PREP and FRCMOD files, compatible with molecular dynamics platforms like AMBER, and caches these for reuse. This efficient approach ensures accurate parameterization while maintaining flexibility for integration into simulation workflows.


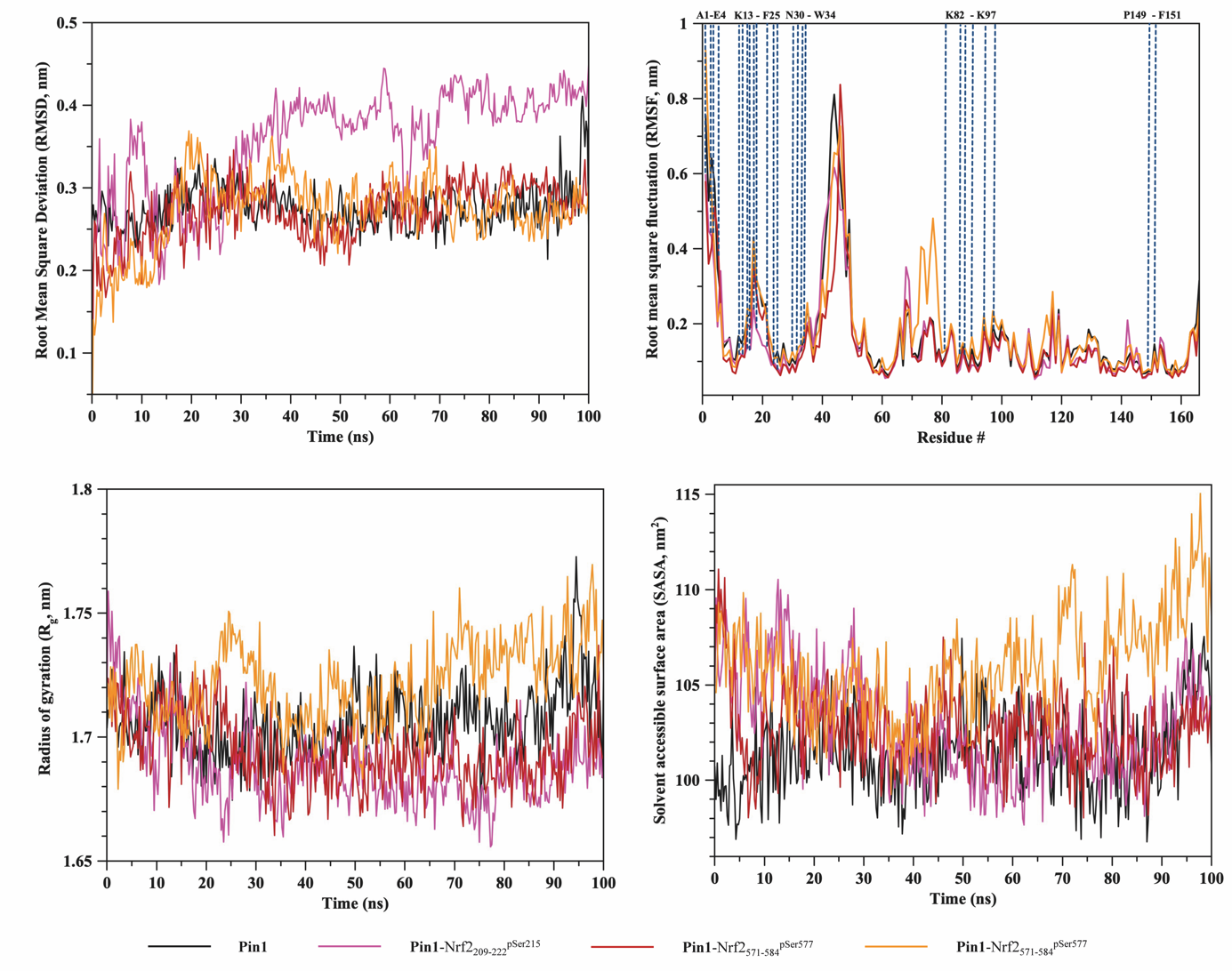


**Supplementary Information Fig. 1:** **RMSD, RMSF, Rg, and SASA graphs for PIN1, PIN1- NRF2_09−222_​^pSer215^, PIN1- Nrf_402−415_​^pSer408^, PIN1- Nrf_571−584_​^pSer577^ proteins.** In RMSF graph, PIN1 interacting residues with NRF2_09−222_​^pSer215^, were indicated with dotted lines. The first and the last residue forming interacting regions were labelled on top of the graph. Detailed explanation of these residues were provided in Figure 2. PIN1 was simulated for a total of 200 ns; however, all analyses were performed on the final 100 ns of the simulations to ensure that the system had reached equilibrium and to enhance the reliability of the results.

The simulation analysis demonstrates how different phosphorylated peptides affect the structural and dynamic properties of PIN1. RMSD results indicate that PIN1 alone is the most stable, with minimal structural deviations. Binding to NRF2_09−222_​^pSer215^ induces the largest structural adjustments, suggesting a strong interaction that significantly alters PIN1’s conformation. RMSF graph cleraly indicated that PIN1 residues that interact with NRF2_09−222_​^pSer215^ became less flexibily upon binding. In specific, the regions between residues Ala 1-Glu 4 and Lys 3-Phe 25, Asn 30-Trp 34 became less flexible upon binding to NRF2_09−222_​^pSer215^, when compared to the same area for PIN1 alone. This indicated a strong interaction and strong binding between PIN1 and NRF2_09−222_​^pSer215^, which was quantified with the calculated free binding energy. The Radius of Gyration (Rg) analysis highlights the differential effects of phosphorylated peptides on the structural compactness of PIN1. PIN1 exhibits stable Rg values, indicating that it maintains its intrinsic compact structure throughout the simulation. When NRF2_09−222_​^pSer215^ and Nrf_402−415_​^pSer408^ are bound to PIN1, PIN1 shows lower Rg values than PIN1 alone. This suggests these two peptides interact with PIN1 which results in changing globular shapes. Likewise SASA analysis indicate same results in Rg analysis NRF2_09−222_​^pSer215^ and Nrf_402−415_​^pSer408^ interacted with PIN1.


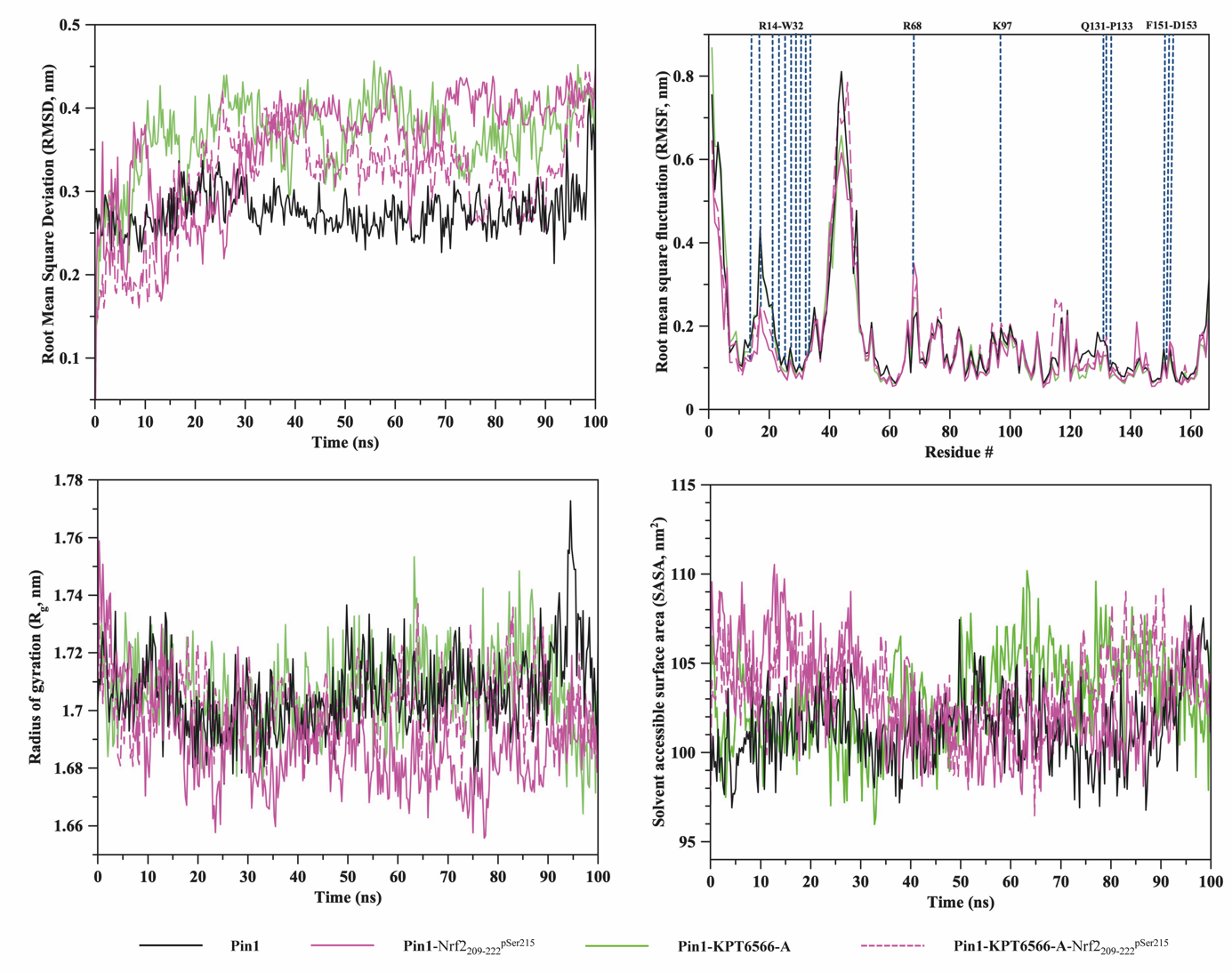


**Supplementary Information Fig. 2: RMSD, RMSF, Rg, and SASA graphs for PIN1, PIN1- NRF2_09−222_​^pSer215^, PIN1-KPT6566-A, PIN1-KPT6566-A-NRF2_09−222_​^pSer215^ proteins.** In RMSF graph, PIN1-KPT6566-A interacting residues with NRF2_09−222_​^pSer215^, were indicated with dotted lines. The first and the last residue forming interacting regions were labelled on top of the graph. Detailed explanation of these residues were provided in Figure 7c.

The analysis revealed that NRF2_09−222_​^pSer215^ bound effectively to PIN1 in the presence of KPT6566-A, inducing structural and dynamic changes on PIN1. RMSD results indicated conformational adjustments caused by KPT6566-A modification, which were amplified by peptide binding, suggesting a synergistic effect. The RMSF graph cleraly showed that the residues of PIN1-KPT6566-A interacting with NRF2_09−222_​^pSer215^ exhibited reduced flexibility upon binding. Specifically, the regions spanning Arg14-Trp32 and Gln131-Phe133 became less dynamic when compared to the same regions in PIN1 alone. This reduction in flexibility indicated a strong interaction and robust binding between PIN1-KPT6566-A and NRF2_09−222_​^pSer215^ as further supported by the calculated binding free energy. Both Rg and SASA analyses showed that KPT6566-A increased PIN1’s structural expansion and solvent exposure, and these effects were further pronounced with NRF2_09−222_​^pSer215^ binding. This reflects peptide-induced structural opening, which likely facilitates functional interactions.


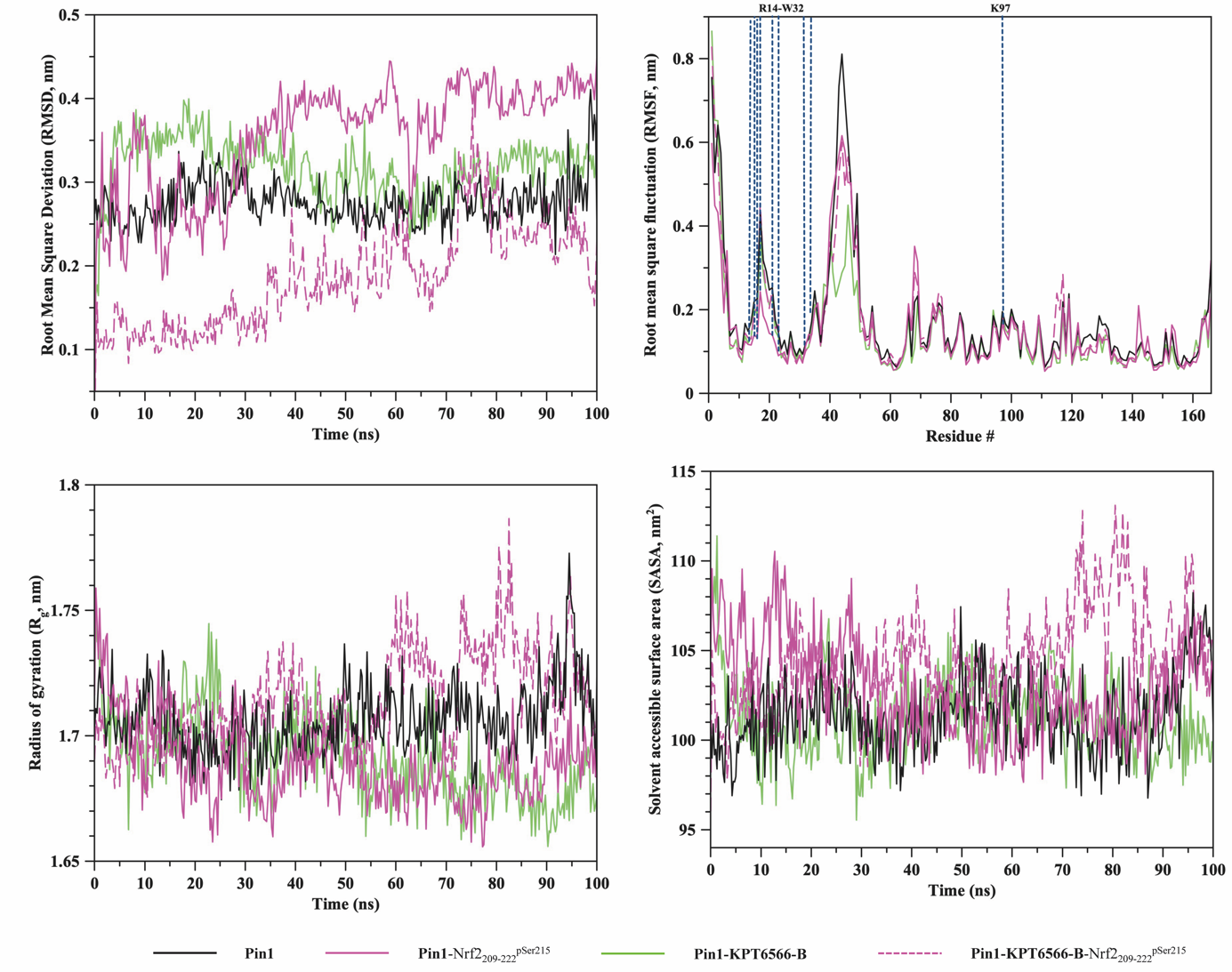


**Supplementary Information Fig. 3: RMSD, RMSF, Rg, and SASA graphs for PIN1, PIN1- NRF2_09−222_​^pSer215^, PIN1-KPT6566-A, PIN1-KPT6566-B-NRF2_09−222_​^pSer215^ proteins.** In RMSF graph, PIN1-KPT6566-B interacting residues with NRF2_09−222_​^pSer215^, were indicated with dotted lines. The first and the last residue forming interacting regions were labelled on top of the graph. Detailed explanation of these residues were provided in Figure 7d.

The RMSD results indicated that while PIN1-KPT6566-B alone underwent structural adjustments due to the covalent modification, the addition of NRF2_09−222_​^pSer215^ further amplified these conformational changes, suggesting a synergistic interaction. RMSF analysis showed reduced flexibility in critical regions, particularly Arg14-Trp32 and Gln131-Phe133, in the PIN1-KPT6566-B-NRF2_09−222_​^pSer215^ complex compared to PIN1 alone, indicating that NRF2_09−222_​^pSer215^ stabilized key residues involved in binding. Rg values demonstrated that KPT6566-B induced a slight expansion of PIN1’s structure, and this effect was further pronounced upon NRF2_09−222_​^pSer215^ binding, reflecting conformational adjustments necessary for accommodating the peptide. Similarly, SASA analysis revealed that KPT6566-B increased solvent exposure of PIN1, and this was further elevated in the presence of NRF2_09−222_​^pSer215^, indicating structural opening and enhanced accessibility.

**REFERENCES:**

1. Schymkowitz, J. *et al.* The FoldX web server: an online force field. *Nucleic Acids Res.* **33**, W382 (2005).

2. Bayly, C. I. *et al.* A Second Generation Force Field for the Simulation of Proteins, Nucleic Acids, and Organic Molecules. *J. Am. Chem. Soc.* **117**, 5179–5197 (1995).

3. Jakalian, A., Jack, D. B. & Bayly, C. I. Fast, efficient generation of high-quality atomic charges. AM1-BCC model: II. Parameterization and validation. *J. Comput. Chem.* **23**, 1623–1641 (2002).

4. YASARA AutoSMILES Server. https://www.yasara.org/autosmilesserver.htm.

5. Wang, J., Wolf, R. M., Caldwell, J. W., Kollman, P. A. & Case, D. A. Development and testing of a general amber force field. *J. Comput. Chem.* **25**, 1157–1174 (2004).

6. Klamt, A. Conductor-like screening model for real solvents: A new approach to the quantitative calculation of solvation phenomena. *J. Phys. Chem.* **99**, 2224–2235 (1995).

7. Bayly, C. I., Cieplak, P., Cornell, W. D. & Kollman, P. A. A well-behaved electrostatic potential based method using charge restraints for deriving atomic charges: The RESP model. *J. Phys. Chem.* **97**, 10269–10280 (1993).
